# Metabolomics on Apple (*Malus domestica*) Cuticle—Search for Authenticity Markers

**DOI:** 10.3390/foods13091308

**Published:** 2024-04-24

**Authors:** Kamila Bechynska, Jiri Sedlak, Leos Uttl, Vit Kosek, Petra Vackova, Vladimir Kocourek, Jana Hajslova

**Affiliations:** 1 Department of Food Analysis and Nutrition, University of Chemistry and Technology, Technicka 3, 16628 Prague 6, Czech Republic; bechynsk@vscht.cz (K.B.); uttll@vscht.cz (L.U.); kosekv@vscht.cz (V.K.); vackovap@vscht.cz (P.V.); kocourev@vscht.cz (V.K.); 2 Reserach and Breeding Institute of Pomology Holovousy, Holovousy 129, 50801 Holovousy, Czech Republic; jiri.sedlak@vsuo.cz

**Keywords:** UHPLC-HRMS/MS, metabolomic fingerprints, classification models, markers, wax esters

## Abstract

The profile of secondary metabolites present in the apple cuticular layer is not only characteristic of a particular apple cultivar; it also dynamically reflects various external factors in the growing environment. In this study, the possibility of authenticating apple samples by analyzing their cuticular layer extracts was investigated. Ultra-high-performance liquid chromatography coupled with high-resolution tandem mass spectrometry (UHPLC-HRMS/MS) was employed for obtaining metabolomic fingerprints. A total of 274 authentic apple samples from four cultivars harvested in the Czech Republic and Poland between 2020 and 2022 were analyzed. The complex data generated, processed using univariate and multivariate statistical methods, enabled the building of classification models to distinguish apple cultivars as well as their geographical origin. The models showed very good performance in discriminating Czech and Polish samples for three out of four cultivars: “Gala”, “Golden Delicious” and “Idared”. Moreover, the validity of the models was tested over several harvest seasons. In addition to metabolites of the triterpene biosynthetic pathway, the diagnostic markers were mainly wax esters. “Jonagold”, which is known to be susceptible to mutations, was the only cultivar for which an unambiguous classification of geographical origin was not possible.

## 1. Introduction

Apple (*Malus* × *domestica* Borkh) is one of the most widely cultivated fruits in the temperate climate zone, with an annual world harvest of around 96 million tonnes [1]. In the Czech Republic, more than 100,000 tonnes of apples were harvested in production orchards in 2023 (based on data from the Central Institute for Supervising and Testing in Agriculture, 2023). Although they are more expensive compared to imported apples, the regional fruits are favored by consumers as they believe that (thanks to the widespread integral farming practices of local producers) fewer pesticides have been used in their production. Unfortunately, under these conditions, dishonest traders tend to falsely declare information when placing this popular fruit on the market. Alongside the intentional false declaration of geographical origin, the other fraudulent practice is the mislabeling of the particular apple cultivar. In detecting such economically motivated frauds, the development of methods to detect them, the aim of which is both to take preventive measures against fraudulent practices and to protect consumers, is a challenging task [2,3].

To date, a variety of instrumental techniques have been published to authenticate the geographical origin and/or cultivar of apples. This includes near-infrared spectroscopy (NIR) [4], fluorescence spectroscopy [5], head-space solid-phase microextraction coupled with gas chromatography and mass spectrometry (SPME-GC-MS) [6,7], isotope ratio mass spectrometry (IR-MS), either separately [2,8,9] or in combination with elemental analysis by inductively coupled plasma mass spectrometry (ICP-MS) [10], and electronic nose and electronic tongue [3]. Further information on these authentication studies is summarized in Table 1. However, as shown here, some of them were performed with a limited number of samples or did not sufficiently cover factors of natural variability (different apple cultivars, growing locations, farming systems, different harvest years, etc.), and in some cases, the description of statistical methods for data processing was insufficient. Under these conditions, generic applicability of the results could be rather difficult.

Various parts and/or processed forms of apples were used within the studies listed in Table 1; nevertheless, only in one of them [4] was the authentication based on the data collected from the apple surface. However, the NIR technique used in that particular study did not allow for the identification of characteristic marker compounds that could be suitable for authentication via targeted analysis. In this context, it is worth noting that the cuticular layer is an interesting matrix in the search for authenticity markers. The cuticular layer contains a number of secondary metabolites whose profile is not only characteristic of the respective cultivar (genotype) but can also be influenced by various external factors in the respective growing location, such as local weather conditions, application of pesticides and growth regulators, diseases and pests. The cuticle, the outer protective layer of the fruit, consists of structural polymers coated with a layer of wax [11]. While the intracuticular waxes are directly incorporated into the cutin, the epicuticular waxes cover a surface of the cutin polymers [12]. The cuticular layer is a complex mixture of secondary metabolites, which include long-chain hydrocarbons and their derivatives, such as carboxylic acids, alcohols, aldehydes and ketones, esters, etc. The other group of typical metabolites are various triterpenoids [13,14,15].

The composition of cuticular waxes has been analyzed in detail in several studies using different analytical methods. For the wax extraction, several authors have used cuticle membranes enzymatically isolated by pectinase and cellulase [14,16]. On the other hand, simple methods based only on rinsing the apple surface with a solvent (usually chloroform, but also dichloromethane, petroleum ether, hexane, etc.) have also been reported [11,17,18,19]. The most common method for analyzing the isolated compounds is GC-MS [11,14,16,18], but this method requires derivatization. Reversed phase liquid chromatography coupled with mass spectrometry (RPLC-MS) has been increasingly used as an alternative, using both atmospheric pressure chemical ionization (APCI) and electrospray ionization (ESI) [20,21,22,23,24,25]. The advantage of LC-ESI-MS is the possibility of the simultaneous detection of different lipid classes, including difficult-to-ionize neutral wax esters when additives are used. The use of high-resolution (HR) MS allows for identification based on molecular or adduct ion exact masse, and the application of collision energy (tandem mass spectrometry, MS/MS) is suitable to obtain information on molecular structure [20].

The aim of the present study was to investigate the potential of UHPL-HRMS-based metabolomic fingerprinting of cuticular layer components, followed by advanced statistics, for the classification of apple cultivars “Gala”, “Golden Delicious”, “Idared” and “Jonagold”, grown either in the Czech Republic or in Poland. To our knowledge, no other published study has used such an approach.

## 2. Materials and Methods

### 2.1. Samples

Apple (*Malus domestica*) samples analyzed in this study were collected by the Research and Breeding Institute of Pomology Holovousy Ltd. (Holovousy, Czech Republic). A total of 274 authentic apple samples of known geographical origin and cultivar characterization were provided over a period of 3 harvest years (2020–2022). The cultivars available in this study were “Gala”, “Golden Delicious”, “Idared” and “Jonagold”, originating either from the Czech Republic (CZE) or Poland (POL). A total of 35 samples of the “Gala” cultivar (16 CZE, 19 POL), 37 samples of the “Golden Delicious” cultivar (19 CZE, 18 POL), 32 samples of the “Idared” cultivar (17 CZE, 15 POL) and 33 samples of the “Jonagold” cultivar (13 CZE, 19 POL) were available. After delivery to the laboratory, the samples were stored at 4 °C for a maximum of 3 days before further processing.

### 2.2. Chemicals

Analytical-grade methanol (MeOH), dichloromethane (DCM), ethyl acetate (EtAC), methyl-tert-butyl-ether (MTBE) and isopropanol (iPrOH) were purchased from Merck (Darmstadt, Germany). Deionized water (dH2O) was obtained from a Milli-Q Integral system (Millipore supplied by Merck (Darmstadt, Germany)). The mobile phase modifiers (ammonium formate, formic acid) were purchased from Sigma-Aldrich (Darmstadt, Germany).

### 2.3. Methods

#### 2.3.1. Sample Preparation

To isolate the metabolites present in the apple cuticular layer, the whole apple was carefully placed into a 1000 mL glass beaker containing 400 mL of solvent mixture. After covering the beaker with parafilm, its content was gently shaken for 10 min. In the experiments undertaken to evaluate the extraction efficiency, the following solvents/mixtures thereof were tested: hexane–EtAc (1:1, *v*:*v*), DCM, DCM–MeOH (2:1, *v*:*v*), MTBE–MeOH (10:3, *v*:*v*) and DCM–MeOH (1:1, *v*:*v*). The last one was selected as optimal and then used throughout the study.

To obtain maximum yield of apple surface components, this procedure was repeated twice, always washing three representative apples. The combined extracts of each sample were evaporated stepwise to dryness and the residue was stored at −80 °C. Prior to analysis, the residue was reconstituted in a calculated amount of DCM:MeOH (1:1, *v*:*v*) to obtain a standardized concentration of the extracted material of 33.33 mg/mL. The solution was filtered using syringe filters (pore size 0.22 μm) and a 1.5 mL aliquot of each extract was then transferred to glass vials for LC-MS analysis. The quality control (QC) sample was prepared as a pool of aliquots of all prepared standardized apple extracts.

#### 2.3.2. UHPLC-HRMS/MS Non-Target Screening

For the metabolomic analysis of apple extracts, the UHPLC-HRMS/MS technique was employed. A high-performance liquid chromatograph Dionex UltiMate 3000 RS (Thermo Fisher Scientific, Waltham, MA, USA) coupled with quadrupole-time-of-flight TripleTOF^TM^ 6600 mass spectrometer (Sciex, Concord, ON, Canada) was used for this purpose. For sample components separation, Acquity UPLC BEH C18 column (2.1 × 100 mm, 1.7 μm) (Waters, Milford, MA, USA) was employed. The mobile phase consisted of (A) 5 mM ammonium formate in a mixture of dH_2_O:MeOH (95:5, *v*:*v*) with 0.1% formic acid and (B) 5 mM ammonium formate in a mixture of iPrOH:MeOH:dH_2_O (65:30:5, *v*:*v*) with 0.1% formic acid. The following gradient was used for both positive and negative ionization modes: 0 min (70% A), 2 min (50% A), 7 min (20% A), 13 min (0% A), 20 min (0% A), 20.1 min (70% A) and 22 min (70% A), with a constant flow rate of 0,4 mL/min. The column temperature was kept at 60 °C; the temperature of the autosampler at 5 °C and the sample injection volume was 1 μL.

The mass spectrometer was operated in both positive (ESI+) and negative (ESI-) mode with the following ion source settings: nebulizing gas pressure, 55 psi; drying gas pressure, 55 psi; capillary voltage, +4500 V (for ESI+)/−4000 V (for ESI-); ion temperature, 500 °C. Both MS and MS/MS data were acquired using full-scan and information-dependent acquisition (IDA) methods. The mass range in MS mode was set to 100–1200 *m*/*z*, and that in MS/MS mode to 50–1200 *m*/*z*. The collision energy was 35 V with the spread of ±15 V. Mass spectrometer calibration was performed regularly after every 10 samples based on APCI calibration solution (Sciex, Concord, Canada).

Samples were injected in a randomized order; QC samples were injected during the entire analytical run (after 10 previous sample injections). Blank samples (extraction solvent mixture) were injected at the beginning of the sequence to capture background features.

#### 2.3.3. Data Processing

The UHPLC-HRMS/MS data obtained were processed using the open-source software MS-Dial (version 4.8) [26]. In the first step, the data were converted into the specific *.ibf format. The peak picking parameters were set as follows for both data acquired in ESI+ and ESI− mode: a minimum signal intensity threshold (peak height) of 10,000; a mass accuracy of 0.01 Da for MS data and 0.025 Da for MS/MS data. For data alignment, retention time tolerance of 0.05 min (ESI+) and 0.3 min (ESI−), along with *m*/*z* tolerance of 0.015 Da, were used. In ESI+ mode [M+H]^+^, [M+Na]^+^ and [M+NH4]^+^ adducts were considered, and in ESI- mode, [M-H]^−^ and [M+HCOO]^−^ adducts were considered.

The exported data matrices, consisting of all detected features characterized by *m*/*z* and retention time, were filtered according to the relative standard deviation (RSD) of the signal intensity (peak area) in the QC samples, with a maximum RSD threshold of 20% for both ESI+ and ESI−. Furthermore, all features with a signal-to-noise ratio (SNR) below 3 were filtered out to obtain the final data matrices.

#### 2.3.4. Statistical Analysis

Prior to statistical analysis, the data were pre-processed to avoid possible misinterpretation of data variability. In this study, the total area sum normalization and logarithmic transformation were performed prior to any univariate statistics, followed by Pareto scaling in the case of multivariate model building.

Within the chemometric processing, the aim was to create models for the classification of apple samples using both univariate and multivariate statistical tools. Principal component analysis (PCA) was used to overview the data. Diagnostics features were selected using *t*-test/analysis of variance (ANOVA), fold change and receiver operating characteristics (ROC) methods. The combination of used methods, where each of them evaluates the feature significance based on a different algorithm, enables the selection of relevant markers. Based on the selected feature subset, both partial least square discriminant analysis (PLS-DA) and orthogonal partial least square discriminant analysis (OPLS-DA) were applied. The developed classification models were validated using 7-fold internal cross validation and characterized by the described variance (R^2^X and R^2^Y), the predicted variance (Q^2^Y), the root mean square error of estimation (RMSEE) and permutation tests for R^2^Y and Q^2^Y.

All statistical analyses were performed using SIMCA^®^ (Sartorius, Göttingen, Germany), Metaboanalyst (metaboanalyst.ca, accessed on 16 January 2024) and using custom built R scripts.

#### 2.3.5. Marker Identification

All significant features (significance is described by the results of the univariate statistical analysis or variable importance on the projection (VIP) score from PLS-DA/OPLS-DA) used for classification models building were subject of structure identification. For these features, *.mat files (containing both MS and MS/MS spectral information) were exported from MS-Dial and imported into the open-source software SIRIUS 4 [27,28], which also integrates CSI:FingerID [29] and CANOPUS [30,31]. Together, these three tools suggest possible molecular formulae, potential structures and compound classes for a given feature, which is compared with online spectral databases (BioCyc, HMDB, COCONUT) [32,33,34]. Tentatively identified markers were characterized by elemental formula, mass error and compound name. In addition, a confidence level of markers identification was classified according to the approaches used in previous studies [35,36,37] for identification of compounds based on LC-MS metabolomic data. The confidence levels range from Level 4 (unknown reproducible signal defined by *m*/*z*, retention time and MS spectrum), Level 3 (known compound class with many isomers possibilities), Level 2 (annotated compound based on matched MS/MS spectra and library) and Level 1 (identified compound confirmed with analytical standard) to Level 0 (identified compound including full stereochemistry).

## 3. Results and Discussion

This study, aimed at the authentication of apple cultivars and their geographical origin, was based on the assumption that characteristic metabolites, authenticity markers, could be identified via the statistical processing of the HPLC-HRMS/MS metabolic fingerprints of cuticular layer extracts. As described in the introduction, several studies have performed well in cultivar or geographical origin classification (Table 1); nevertheless, none of them focused on the analysis of the cuticular layer extracts, which, according to our working hypothesis, have a high application potential for authentication. In the paragraphs below, the steps taken to test this working hypothesis are described.

### 3.1. Selection of Extraction Solvent/Mixture

The first step was to find an extraction solvent that would enable the reproducible extraction (not necessarily quantitative) of the widest possible range of substances from the apple cuticle. The tested solvents/solvent mixtures, differing in their selectivity, involved hexane–EtAc (1:1, *v*:*v*), MTBE–MeOH (10:3, *v*:*v*), DCM, DCM–MeOH (2:1, *v*:*v*) and DCM–MeOH (1:1, *v*:*v*). The comparison of the total ion chromatograms of the tested solvents is shown in Supplementary Materials (Figure S1). The suitability of the extraction solvent was assessed by the distribution of chromatographic peaks in terms of their retention times and the total number of features detected via the reversed-phase UHPLC-HRMS method, which is commonly used by the authors of metabolomic studies. Since wax esters, which are known to occur in large amounts in the apple cuticular layer [13], ionize poorly in ESI- mode, only ESI+ was used for the experiments. A similar approach was used in other studies analyzing a similar matrix [20,22]. Based on the above criteria, the best solvent mixture was DCM–MeOH (1:1, *v*:*v*). A total of 11,581 features corresponding to compounds with a wide range of polarities were detected. Only a slightly lower number of features were detected in DCM and DCM–MeOH (2:1, *v*:*v*) extracts, while in the chromatograms obtained with the other solvent mixtures, hexane–EtAc (1:1, *v*:*v*) and MTBE–MeOH (10:3, *v*:*v*), the more polar metabolites, eluted at lower retention times, were not sufficiently represented.

### 3.2. UHPLC-HRMS/MS Analysis

**Figure 1:**
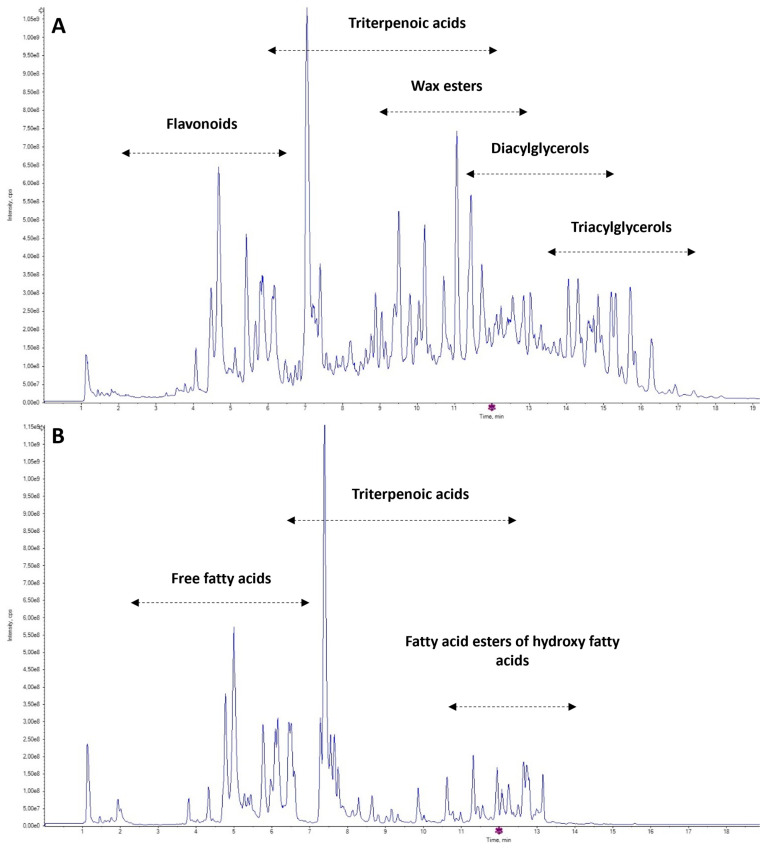
Total ion chromatogram of apple cuticle layer extract, QC sample: (**A**) ESI+ mode and (**B**) ESI− mode.

A number of analytical strategies have been applied for the investigation of apples’ authenticity, either geographically or by variety. As shown in Table 1, in most cases, non-target screening performed by various instrumental techniques, such as NIR, GC-MS or IR-MS, was employed for analyses of various apple parts/forms. In contrast to these approaches, the UHPLC-HRMS/MS technique used in this study allows for not only the acquisition of the metabolomic fingerprints of the respective sample but also the identification of diagnostic markers (without pre-analytical derivatization) that can be used for authentication based on a simpler target screening applicable under routine conditions. The complexity of the apple cuticular layer extract is documented by the total ion chromatograms (TIC) in Figure 1A,B. As expected, wide range of lipophilic compounds was isolated; their main groups are indicated in the figures.

### 3.3. Chemometric Analysis

Processing of all raw UHPLC-HRMS/MS data with MS-Dial software resulted in the detection of 96,072 features in positive ionization mode and 21,040 features in negative ionization mode. Both of the aligned data matrices obtained were then filtered according to the criteria mentioned in Materials and Methods (Section 2.3.3) based on RSD in the QC sample (20%) and the SNR in the blank sample (≥3). As a result of feature filtering, the final data matrices contained 16,044 features and 2132 features, respectively, in ESI+ and ESI- mode, respectively. These data were used for further processing.

#### 3.3.1. Data Overview

**Figure 2:**
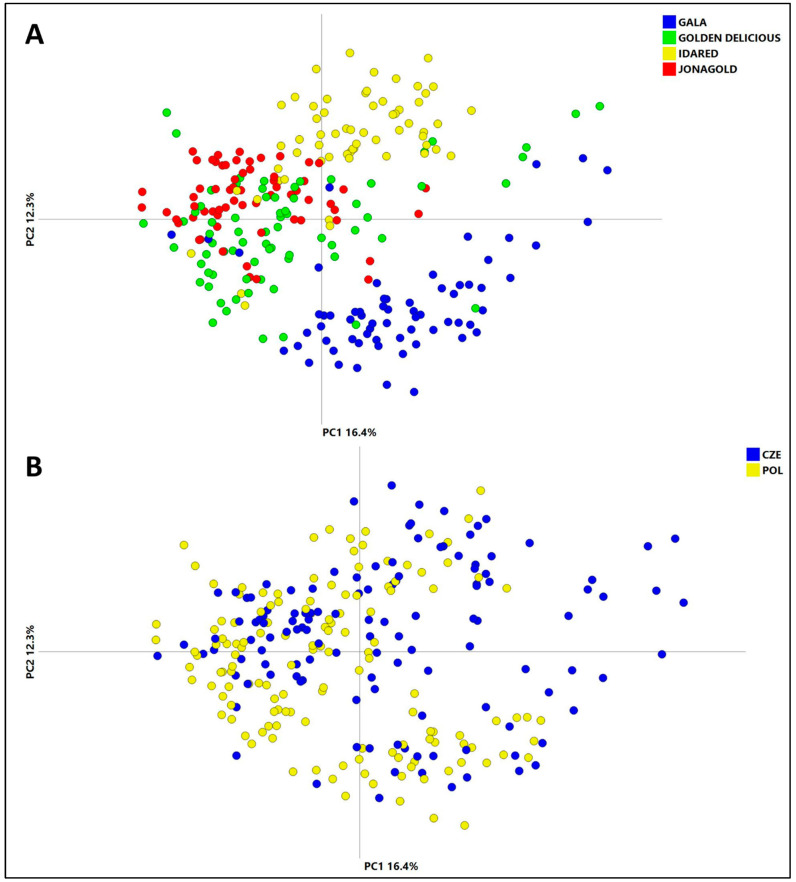
PCA Score plots of the complete dataset of ESI+ features. Samples are colored according to (**A**) their cultivar and (**B**) their geographical origin.

Data visualization via PCA revealed a clustering of the samples based on cultivar (Figure 2A). However, the separation of apples from the Czech Republic and Poland by geographical origin was not pronounced (Figure 2B). The data shown in these figures were obtained for the ESI+ mode; similar trends are documented in the PCA Score plots for the ESI− mode (see Supplementary Materials Figure S2).

In order to obtain reliable models for the classification of both apple cultivars and geographical origin, two alternative methods of supervised learning were combined. PLS-DA was used for cultivar classification, while a systematic strategy was used for geographical origin classification, as the influence of growing location on the metabolome of the cuticular layer is less pronounced. Individual binary OPLS-DA models were created for each apple cultivar. The use of this specific chemometric method allowed us to reduce the effect of collinearity and model overfitting [38].

#### 3.3.2. Apple Cultivars Classification

Prior to building the PLS-DA classification models, an ANOVA false discovery rate (FDR) *p*-value threshold of 0.05 was used to filter out features that were unimportant for cultivar differentiation. This process resulted in a subset of 14,551 and 1678 significant features for the ESI+ and ESI- modes, respectively, which were used for building PLS-DA models (all four groups of cultivars included). For the model based on ESI+ data, the validation parameters were R^2^Y = 0.790 and Q^2^Y = 0.756; for the ESI- data, similar qualitative results were achieved, with R^2^Y = 0.770 and Q^2^Y = 0.756. Permutation tests (*n* = 100) were performed to validate the developed models; for both R^2^Y and Q^2^Y, the *p*-value was below 0.01, indicating valid models [39]. In general, a VIP score > 1 is considered as a threshold value for significant features [40]; nevertheless, in the study here presented, this threshold was increased up to 1.5, which resulted in exclusion of less significant metabolites. This way, only the 197 most significant markers (ESI+ and ESI− combined) were selected for further processing. Table 2 provides an overview of 13 metabolites whose molecular structure could be identified using the criteria specified in Materials and Methods (Section 2.3.5).

**Figure 3:**
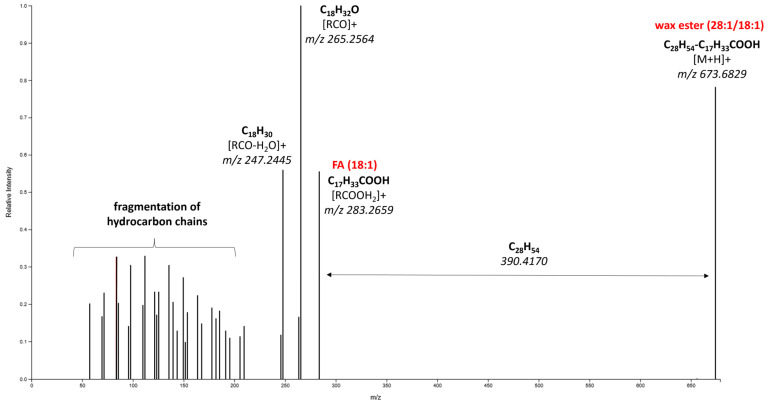
Fragmentation spectrum of marker identified as wax ester (28:1/18:1).

Flavonoids, wax esters and other lipids together with triterpenoids were the three classes of compounds identified as markers. The first group included isorhamnetin (methylated metabolite of quercetin), isorhamnetin rhamnoside and luteolin malonyl glucoside, secondary metabolites reported to be present at low levels in apple skin [41,42]; all were present at higher levels in “Idared” and “Jonagold” apple cultivars. The second group of significant markers (according to the VIP score) was identified as wax esters, a lipid subclass defined by the LIPID MAPS structure database (LMSD) [43]. Although many studies have indicated the presence of wax esters in the apple cuticular layer [12,44,45,46], the (tentative) identification of individual representatives relied exclusively on gas chromatography coupled to a flame ionization detector or mass-spectrometry with a simple mass analyzer, where the sample preparation typically involved the hydrolysis of ester bonds and a derivatization step aimed at increasing the volatility of the released fatty acids and alcohols under these conditions; some of the information about the wax structure is lost. Contrary to that approach, in another earlier comprehensive study [47], GC-MS analysis of whole molecules was performed; a database involving electron ionization mass spectra of 154 wax ester standards (various straight-chain and methyl-branched saturated and unsaturated species) was created. As regards LC-MS, several authors have investigated the mass spectra of wax esters obtained via this technique in more detail. In two older studies [48,49], atmospheric pressure ionization (APCI) and two types of mass analyzers (ion trap and Orbitrap) were used for this purpose. The dominant signals in the mass spectra were protonated molecular ions [M+H]^+^. Also in our study, where ESI was used instead of APCI, the most intensive ions in the wax ester spectra were [M+H]^+^. On the other hand, some papers report the use of ESI (and ammonium formate was a component of a mobile phase), but the most intensive adducts of wax esters were [M+NH_4_]^+^ [50,51]. In our study employing the Q-ToF mass analyzer, the fragmentation spectra of the [M+H]^+^ ion were further investigated and compared with those reported by Chen et al. [20], who focused on a systematic investigation of collision-induced dissociation (CID) patterns of different wax ester standards using HPLC-ESI-Q-ToF-MS. Based on this information, the fragmentation spectra of some wax esters present in our samples could be interpreted; an example of one of identified markers as wax ester (28:1/18:1) is shown in Figure 3.

The molecular formula C_46_H_88_O_2_ was calculated for the precursor ion *m*/*z* 673.6829 based on its exact mass and isotopic envelope. In the MS/MS spectrum, the base ion *m*/*z* 283.2659 was identified as an octadecenoic acid fragment [RCOOH_2_]^+^, which was formed by breaking the ester bond in the wax molecule. The fragments ions *m*/*z* 256.2564 and *m*/*z* 247.2445 were residues of oleic acid, namely, [RCO]^+^ and [RCO-H_2_O]^+^, respectively, which is in line with the spectra interpretation introduced by the aforementioned study [20]. The calculated difference between the precursor ion [M+H]^+^ (*m*/*z* 673.6829) and the fatty acyl fragment (*m*/*z* 283.2659) 390.4170 corresponded to the elemental composition C_28_H_54_, i.e., the loss of the octacosenol alkyl moiety. The fragmentation spectra for other wax esters with identified fatty acid and fatty alcohol moieties are shown in File S1. The description of the wax esters’ structure (number of carbon atoms in the alcohol; number of double bonds/number of carbons in the fatty acid; number of double bonds) corresponds to the nomenclature used in LMSD for this lipid subclass [52]. Another identified marker belonging to lipids was the fatty acid ester of hydroxy fatty acid (FAHFA, 18:1/16:1). These FAHFAs (previously known as indicators of inflammation in human samples) have only recently been quantified in some foods, including apple skin [53]. The other identified markers representing the class of acylglycerols were DAG (46:7), DAG (28:2) and TAG (60:2).

The last group of identified markers of apple cultivar were triterpenic acids, a group of compounds that was the only one analyzed in the apple cuticle by several authors using the UHPLC-HRMS/MS technique [22,23,25]. Caffeoylbetulinic acid, one of the tentatively identified triterpenic acids, was, together with caffeoyloleanolic acid, recognized earlier as a typical metabolite occurring in the skins of russeted apples [25]. It should be noted that susceptibility to russeting is rather cultivar-specific; some apples, such as Idared, develop this defect only very rarely.

It must be emphasized that despite a relatively small number of markers used for cultivars classification, the performance characteristics of the PSL-DA model were acceptable with R^2^Y = 0.691, Q^2^Y = 0.666 and recognition ability of 91% [39]. The boxplots illustrating markers can be found in Supplementary Materials (Figure S3).

#### 3.3.3. Classification of Apple Geographical Origin

The comparison of the PCA cosre plots (Figure 2 and Figure S1) documents a bigger impact of the apple cultivar on the fingerprint of the cuticular-layer metabolome than that of the geographical origin. For this reason, the generated data were investigated more in depth and separately for each variety. The input file was a filtered data matrix with 16,044 and 2132 features, obtained in ESI+ and ESI− mode, respectively. Both *t*-test and ROC were then applied to the data and the fold change was calculated. An OPLS-DA model was created based on the subset of features that met the *t*-test FDR *p*-value < 0.05 and where the area under curve (AUC) value was higher than 0.75 [54]. Apart from the model validation performed, which was performed using seven-round cross-validation [55], the validity of the model was also proved over several harvest seasons. The predictive ability of the model was calculated by inserting samples from the 2020 and 2022 harvest seasons into the model created from samples harvested in 2021. The performance characteristics of all created OPLS-DA models for all cultivars are summarized in Table 3.

As shown in Table 3, all OPLS-DA models, except for the one classifying the geographical origin of “Jonagold” apples (ESI− data), performed satisfactorily [39]. The highest number of features differentiating between apples from Poland and the Czech Republic was found for the “Golden Delicious” cultivar. On the other hand, the worst performance of the developed classification models was found for the “Jonagold” cultivar, which is known to be susceptible to mutations (according to the experts from the Czech Research and Breeding Institute of Pomology Holovousy, there are 23 known mutations in “Jonagold” compared to 4 known mutations in “Golden Delicious”, 3 in “Idared” and 8 in “Gala”). Rather high variability of apple metabolite patterns in the case of “Jonagold” cultivar (even within the respective country) was obviously associated with its high susceptibility to mutations. For this reason, it was difficult to identify reliable diagnostic markers. When searching for experiences with the authentication of “Jonagold” in other studies, we found that Chinese authors [6] included this variety in their sample set, together with “Starkrimson”, “Qinguan”, “Gala”, “Golden Delicious” and “Fuji”. However, a different approach, the fingerprinting of volatiles in apple juices via the SPME-HS-GC-MS technique, was used by authors. Interestingly, contrary to other cultivars, the authentication of which was 100% successful, in the case of Jonagold, it was only 89%.

All features selected for geographical origin classification (distinguishing between Czech and Polish apple samples), i.e., those with ROC AUC value > 0.75 and *t*-test FDR *p*-value < 0.05, were subjected to the identification process. A total of, 40, 72 and 6 markers were identified for “Gala” (Table S1), “Golden Delicious” (Table S2) and “Idared” 6 (Table S3) cultivars, respectively. With regard to the facts mentioned above, no marker was identified for “Jonagold” cultivar.

**Figure 4:**
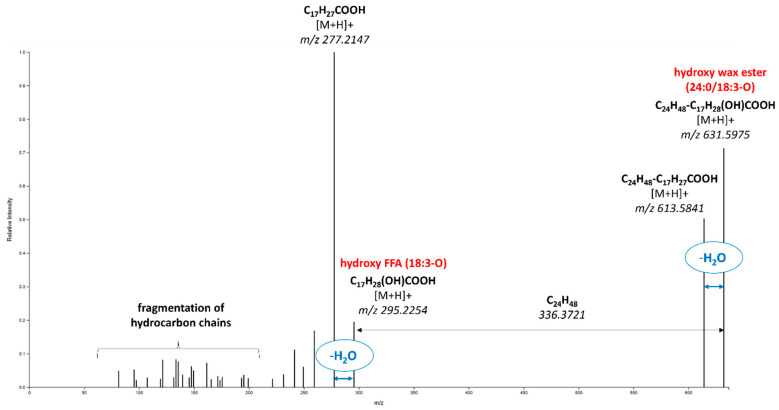
Fragmentation spectra of the marker identified as hydroxy wax ester (24:0/18:3-O).

**Figure 5:**
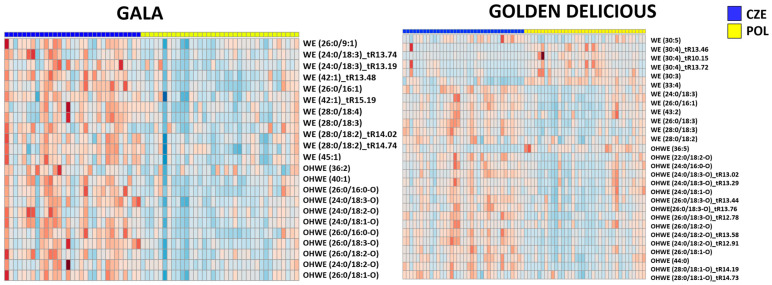
Heatmaps showing fold changes (red and blue colours represent positive and negative fold changes, respectively, darker shadows indicate higher absolute values) of wax ester (WE) and hydroxy wax ester (OHWE) intensities corresponding to the median value of the respective compound in apple samples (on the horizontal axis) harvested either in the Czech Republic (blue) or Poland (yellow).

The most frequent markers for geographical origin were wax esters, 23 of them for “Gala” and 28 for “Golden Delicious”. In addition to fatty acids, hydroxy fatty acids were also bound in wax ester molecules. The example of the fragmentation spectrum of such a marker (*m*/*z* 631.5975; retention time: 13.02 min), which was identified as a hydroxy wax ester (24:0/18:3-O), is shown in Figure 4. The obtained spectra were similar to those of the wax esters discussed in the previous section, but the calculated molecular formula (in this case, C_42_H_78_O_3_) contained three oxygen atoms instead of two. In the fragmentation spectra of these compounds, alike to that shown in Figure 5, there was a visible neutral loss of H_2_O molecules from both precursor and fragment ions corresponding to the hydroxy fatty acyl [56]. A total of 27 hydroxy wax esters were identified as markers of geographical origin in the analyzed apples. For those, in which fatty acid composition was tentatively identified, the corresponding fragmentation spectra are summarized in File S1.

In general, the most common fatty acids bound in waxes were hexadecanoic acid, hexadecenoic acid (16:0, 16:1) and unsaturated fatty acids containing 18 carbons (18:1, 18:2, 18:3, 18:4) bound to C22-C28 aliphatic alcohol. The heatmaps in Figure 5 show that in both “Gala” and “Golden Delicious” cultivars, the wax esters, as well as hydroxy wax esters, were upregulated in the apples harvested in the Czech Republic (exceptions were shorter chain wax esters, i.e., 30:3, 30:4, 30:5). In “Gala”, the differences were more pronounced than in “Golden Delicious”; the fold change of wax esters ranged from 1.21 to 2.37 and from 1.11 to 3,17, respectively. The higher intensities of wax esters signals (i.e., their higher amounts) observed in the samples from the Czech Republic could be due to the higher average altitude (and thus the different climatic conditions) of the orchards where the apples were collected (450 m in the Czech Republic versus 173 m in Poland), which has been previously reported as a parameter associated with thicker cuticles [57].

The next group of identified geographical origin markers were triterpenic acids, specifically ursane-type triterpenic acids. As already mentioned, these compounds, together with their derivatives (oxo, dihydroxy, oxohydroxy), have been identified in the apple cuticle layer by several authors [22,23].

In the case of our data, eight derivatives of ursolic acid were identified among the markers for the cultivar “Gala”. Interestingly, all ursenoic acids showed increased intensity in Czech apple samples. The higher intensity was statistically significant based on *t*-test *p*-value < 0.05 (except for oxohydroxy ursenoic acid), with fold changes ranging from 1.3 to 4. In “Golden Delicious”, in addition to the five ursolic acid derivatives, their precursors lupeone, uvaol, amyrin and hydroxybetulin were also found. Among markers for cultivar “Golden Delicious”, there was no clear trend. As an example, the boxplots of all ursenoic acids selected as markers are shown in the Supplementary Materials (Figures S4 and S5).

As relatively polar metabolites such as sorbitol, fucose, heptulose and mannose (trace amounts were contained in analyzed extracts), alike some flavonoids, represented by phloretin and chlorogenic acid common apple flavonoids [58,59], were also on the list of geographic origin markers.

## 4. Conclusions

In this study, the UHPLC-HRMS/MS-based metabolomic fingerprinting of cuticular layer extracts followed by advanced data processing was proved to be an applicable strategy for the authentication of apples cultivars and their geographic origin. The results of this study, within which 274 apple samples of four cultivars harvested either in the Czech Republic or Poland in three seasons were analyzed, can be summarized as follows:

- PCA showed a more pronounced cultivar impact on the metabolites occurring in the apple cuticle compared to that of geographical origin.
- The created PLS-DA models enabled reliable apple cultivar classification; 13 markers encompassing mainly waxes and triterpenoids were identified,
- The created OPLS-DA models enabled the safe classification of geographical origins of “Gala”, “Golden Delicious” and “Idared” cultivars; however, for “Jonagold”, it was unsuccessful.
- Wax esters, including those with bound hydroxy fatty acids (reported for the first time in apple cuticular wax), represented a significant group of identified markers, the amount of which in “Golden Delicious” and “Gala” cultivars was higher (upregulated) in samples from the Czech Republic compared those from Poland.

Overall, our findings underscore the potential of apple cuticular layer analysis to be used as a robust tool for apple authentication, offering insights into both cultivar and geographical origin distinctions. Moreover, revealing novel compounds that enhance our understanding of apple wax composition is facilitated by this approach.

## Figures and Tables

**Table 1:** The overview of studies dealing with the authentication of the origin and/or cultivar of apples.

Analytical Method	Description of Apple Samples	Classification Factor	Number of Samples	Number of Classes to Be Distinguished within the Sample Set	Classification Method	Performance of Classification	Reference
NIR	Surface of whole apple fruits	Cultivar	300	3 (Fuji, Red Star, Gala)	NN, SVM, ELM	Calibration set 98% (ELM) Prediction set 97% (ELM)	[4]
		Geographical origin		2 (grown in different Chinese provinces)			
Fluorescent spectroscopy	Apple juice (squeezed with a juice extractor)	Cultivar	89	2 (grown in different Chinese provinces)	PLS	Calibration set 100% Prediction set 96%	[5]
SPME-GC-MS	Apple juice (squeezed with a juicer)	Cultivar	50	6 (Starkrimson, Qinguan, Gala, Jonagold, Golden Delicioius, Fuji)	LDA, SLDA	Predicition set 100% (SLDA)	[6]
		Geographical origin		5 (grown in different counties within Chinese province)		Predicition set 90% (SLDA)	
SPME-GC-MS	Apple juice (squeezed with hand press)	Cultivar	4 (3 kg of apples per sample)	4 (Rijo, Verde, Ribeiro, Azedo)	PLS-DA, HCA	Vague description of model performance	[7]
		Geographical origin		2 (different civil parishes of Madeira)			
IR-MS + conventional methods	Pulp, juice	Cultivar	19	6 (Topaz, Idared, Golden Delicious, Goldrush, Gala, Gloster)	LDA	Insufficient description of models	[2]
		Geographical origin		4 (different regions of Slovenia)			
		Agricultural practice		2 (way of farming organic, conventional)			
IR-MS	Whole apples, peel, pulp, seed	Cultivar	128	4 (Cripps Pink, Gala, Golden Delicious, Granny Smith)	LDA	71% correctly classified samples	[8]
		Geographical origin		4 (grown in different districts of northerm Italy)		99% (LOOCV)	
IR-MS	Peel, petiole, pulp, seed	Geographical origin	48	2 (grown in different districts of northern Italy)	LDA	Limited information on classification models performance	[9]
IR-MS, ICP-MS	Apple juice (concentrated to sugar content 65.0°Brix)	Geographical origin	135	6 (grown in different Chinese provinces)	LDA, PLS-DA	Only description of sample clustering in PLS-DA model without information about model validation	[10]
Electronic nose, electronic tongue	Apple juice (centrifugal juicer)	Cultivar	126	10 (Fuji, Jonagold, Corolla, Gala, Red Delicous, Red Chief Delicious, Cattle Apple, Ralls Janet, Ourin, Tail, Golden Delicous)	LDA, PLS-DA, SVM	100% (prediction ability of PLS-DA) 100% (accuracy testing rate of SVM)	[3]
		Geographical origin		7 (grown in different Chinese provinces)			

ELM—extreme learning machine; HCA—hierarchical cluster analysis; LDA—linear discriminant analysis; LOOCV—leave-one-out cross validation; NN—neural networks; PLS—partial least square; PLS-DA—partial least square discriminant analysis; SLDA—stepwise linear discriminant analysis; SVM—support vector machine.

**Table 2:** Identified significant metabolites used for apple cultivar classification (compounds sorted in descending order according to the PLS-DA VIP score).

Marker Ion (*m*/*z*)	Retention Time [min]	Adduct Type	Elemental Formula	Mass Error [ppm]	Tentative Identification	PLSDA VIP Score	Confidence Level
701.7138	14.03	[M+H]+	C_48_H_92_O_2_	−5.4	Wax ester (30:1/18:1)	3.1	2
317.064	2.06	[M+H]+	C_16_H_12_O_7_	−6.7	Isorhamnetine	2.9	3
673.6829	13.69	[M+H]+	C_46_H_88_O_2_	−5	Wax ester (28:1/18:1)	2.9	2
461.1111	2.14	[M-H]−	C_22_H_22_O_11_	5.9	Isorhamnetin rhamnoside	2.8	3
671.6652	13.43	[M+H]+	C_46_H_86_O_2_	-8	Wax esters (46:3)	2.8	3
699.691	14.64	[M+Na]+	C_46_H_92_O_2_	−12.1	Wax esters (46:0)	2.7	3
979.8971	14.81	[M+Na]+	C_63_H_120_O_5_	−6.4	TAG (60:2)	2.4	3
509.4234	12.24	[M+H]+	C_31_H_56_O_5_	5.6	DAG (28:2)	2.1	3
533.0917	1.33	[M-H]−	C_24_H_22_O_14_	2.6	Luteolin-O-malonyl glucoside	2.1	3
663.3906	5.99	[M+HCOO]−	C_39_H_54_O_6_	1.3	Caffeoylbetulinic acid	2	3
535.4747	12.87	[M-H]−	C_34_H_64_O_4_	3.8	FAHFA (18:1/16:0)	1.9	2
549.3436	3.7	[M+HCOO]−	C_30_H_48_O_6_	1.6	Triterpenic acid	1.6	3
749.6105	13.21	[M-H]−	C_49_H_82_O_5_	2.8	DAG (46:7)	1.6	3

DAG—diacylglycerol; FAHFA—fatty acid ester of hydroxy fatty acid; PLSDA VIP—variable importance in projection of PLSDA model; TAG—triacylglycerol

**Table 3:** Parameters of the OPLS-DA models for the classification of apple samples according to the geographical origin (Poland vs. Czech Republic).

OPLS-DA Model Parameters	ESI+				ESI−			
	Gala	Golden Delicious	Idared	Jonagold	Gala	Golden Delicious	Idared	Jonagold
number of features	506	1048	156	11	13	44	24	9
R^2^X	0.783	0.596	0.570	0.946	0.667	0.567	0.850	0.921
R^2^Y	0.735	0.635	0.886	0.561	0.639	0.738	0.646	0.480
Q^2^Y	0.624	0.554	0.809	0.501	0.543	0.686	0.574	0.436
RMSEE	0.265	0.309	0.175	0.335	0.307	0.261	0.308	0.362
*p*-value of permutation for R^2^Y	<0.01	<0.01	<0.01	<0.01	<0.01	<0.01	<0.01	<0.01
*p*-value of permutation for Q^2^Y	<0.01	<0.01	<0.01	<0.01	<0.01	<0.01	<0.01	<0.01
validity of the model over time	82%	65%	85%	88%	78%	77%	78%	88%

R^2^X—fraction of X variation described by the model; R^2^Y—fraction of Y variation described by the model; Q^2^Y—fraction of Y variation predicted by model according to the cross validation; RMSEE—root mean square error of estimation.

## Data Availability

The original contributions presented in the study are included in the article and Supplementary Materials, further inquiries can be directed to the corresponding author.

## Supplementary Materials

The following supporting information can be downloaded at https://www.mdpi.com/article/10.3390/foods13091308/s1: Figure S1: Total ion chromatograms (ESI+) showing intensities of compounds extracted by various solvent mixtures. Figure S2: PCA Score plots of the complete dataset of ESI− features. Samples are colored according to their cultivar (A) and geographical origin (B). Figure S3: Boxplots of identified markers for classification of apple cultivar. Figure S4: Boxplots of markers for classification of geographical origin for cultivar “Gala” identified as ursane-type triterpene acids and their derivatives and precursors. Figure S5: Boxplots of markers for classification of geographical origin for cultivar “Golden Delicious” identified as ursane-type triterpene acids and their derivatives and precursors. Table S1: Identification of metabolites used for geographical origin classification of apple cultivar “Gala”. Markers are in descending order according to the AUC ROC value. The log2 FC value indicates whether the marker is increased in Czech samples (log2 FC > 0) or in Polish samples (log2 FC < 0). Table S2: Identification of metabolites used for geographical origin classification of apple cultivar “Golden Delicious”. Markers are in descending order according to the AUC ROC value. The log2 FC value indicates whether the marker is increased in Czech samples (log2 FC > 0) or in Polish samples (log2 FC < 0). Table S3: Identification of metabolites used for geographical origin classification of apple cultivar “Idared”. Markers are in descending order according to the AUC ROC value. The log2 FC value indicates whether the marker is increased in Czech samples (log2 FC > 0) or in Polish samples (log2 FC < 0). File S1: Fragmentation spectra of all wax esters with identified fatty acid and fatty alcohol moiety.
