# Supplementary material for: Metabolomics on Apple (Malus domestica) Cuticle—Search for Authenticity Markers
Source: Foods. 2024 Apr 24;13(9):1308. doi: 10.3390/foods13091308 (PMC11083494; doi:10.3390/foods13091308)
Supplement: Supplementary file 1 [file foods-13-01308-s001.zip › File S1.pptx]

## Slide 1
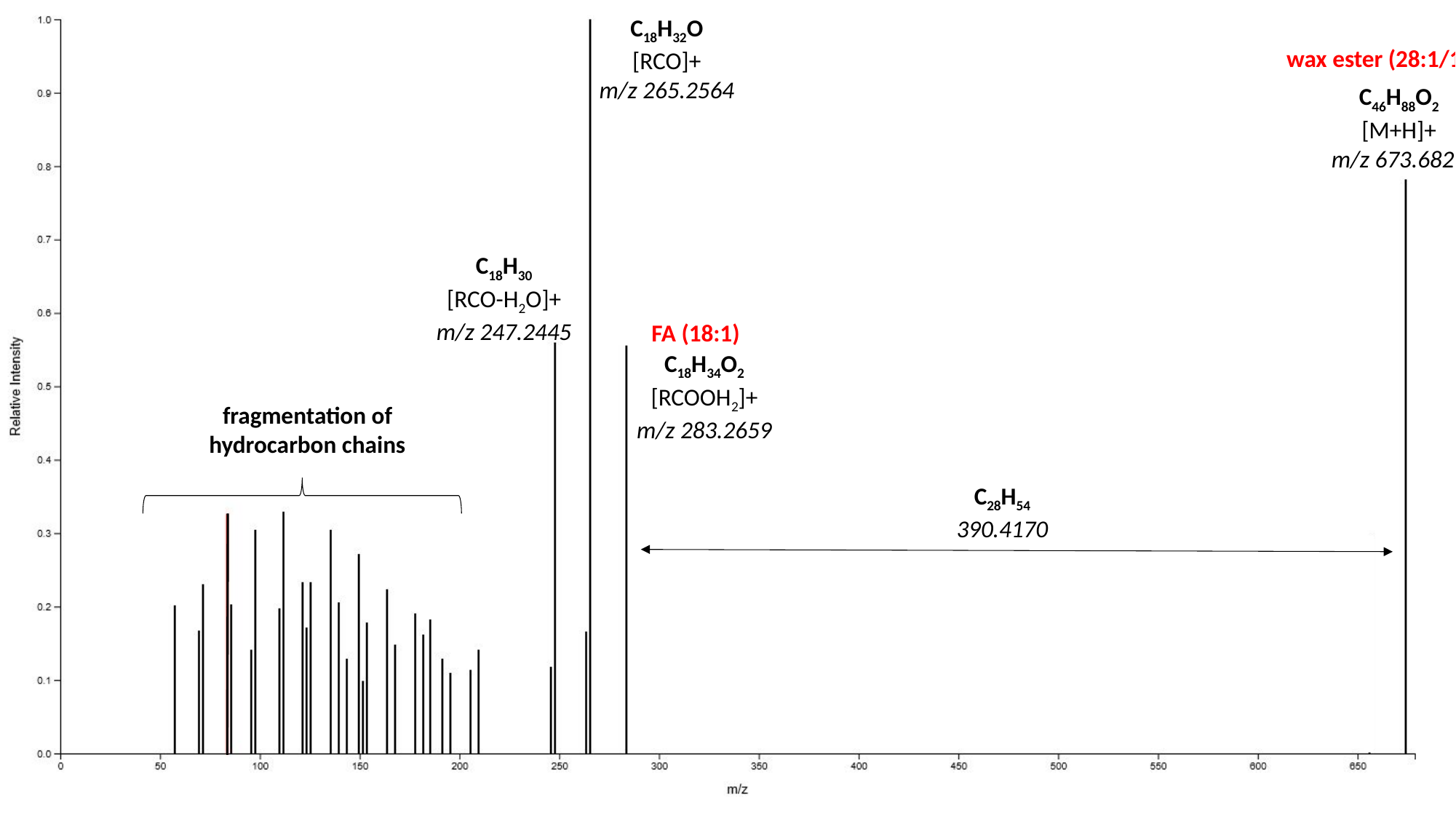

C18H32O
[RCO]+
m/z 265.2564
wax ester (28:1/18:1)
C46H88O2
[M+H]+
m/z 673.6829
C18H30
[RCO-H2O]+
m/z 247.2445
FA (18:1)
C18H34O2
[RCOOH2]+
m/z 283.2659
fragmentation of hydrocarbon chains
C28H54
390.4170

## Slide 2
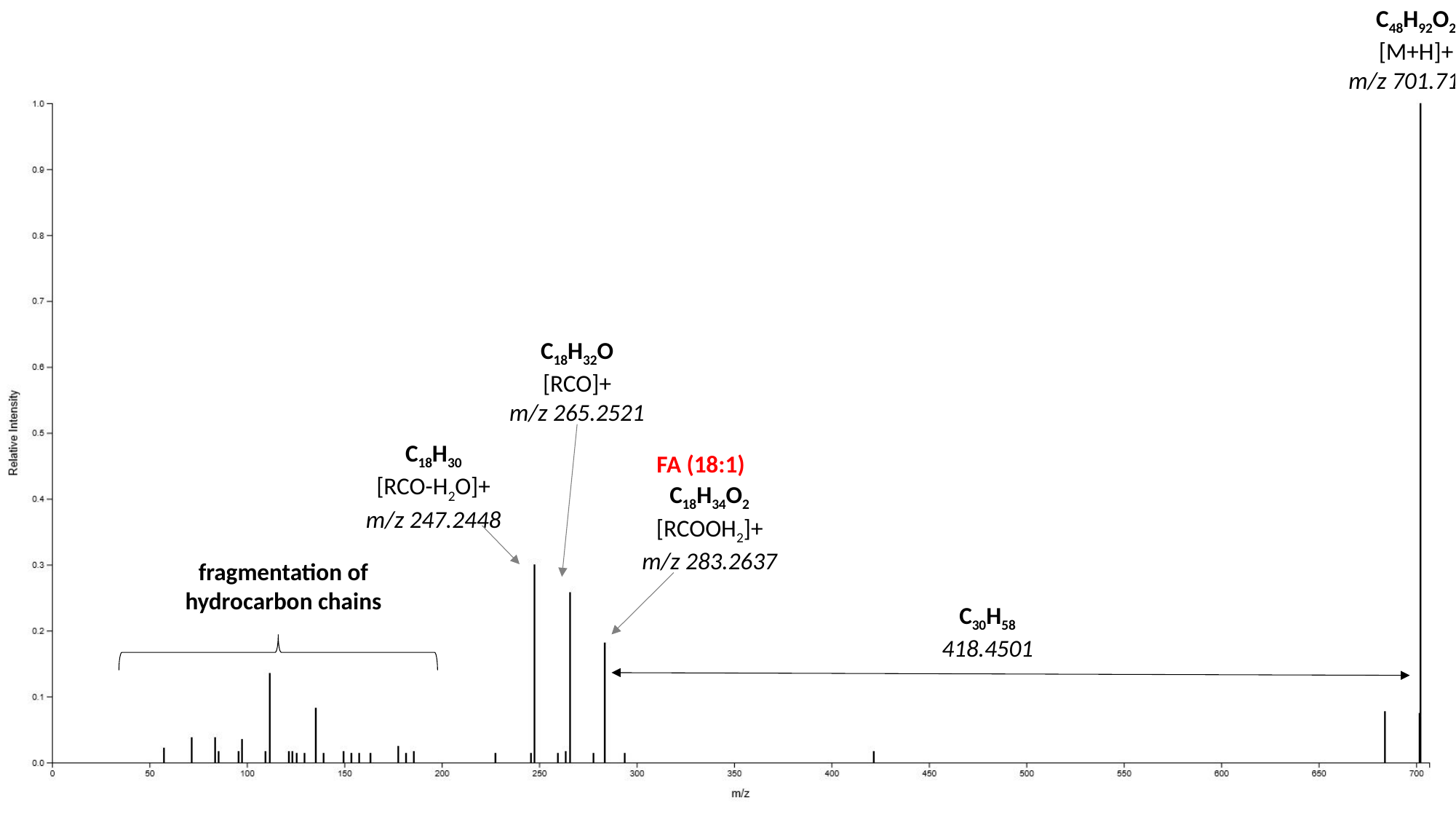

wax ester (30:1/18:1)
C48H92O2
[M+H]+
m/z 701.7138
C18H32O
[RCO]+
m/z 265.2521
C18H30
[RCO-H2O]+
m/z 247.2448
FA (18:1)
C18H34O2
[RCOOH2]+
m/z 283.2637
fragmentation of hydrocarbon chains
C30H58
418.4501

## Slide 3
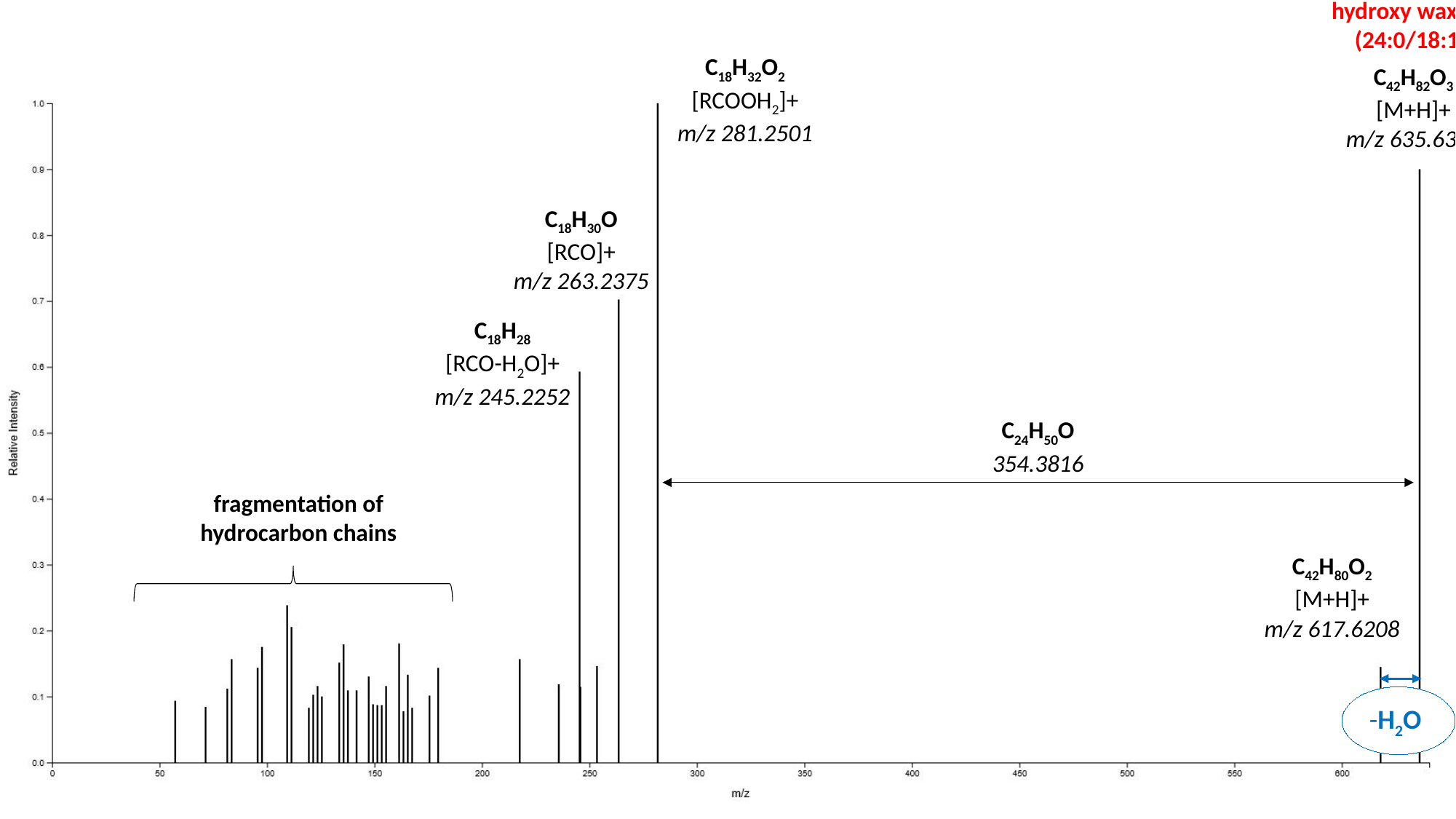

hydroxy wax ester (24:0/18:1-O)
C18H32O2
[RCOOH2]+
m/z 281.2501
C42H82O3
[M+H]+
m/z 635.6317
C18H30O
[RCO]+
m/z 263.2375
C18H28
[RCO-H2O]+
m/z 245.2252
C24H50O
354.3816
fragmentation of hydrocarbon chains
C42H80O2
[M+H]+
m/z 617.6208
-H2O

## Slide 4
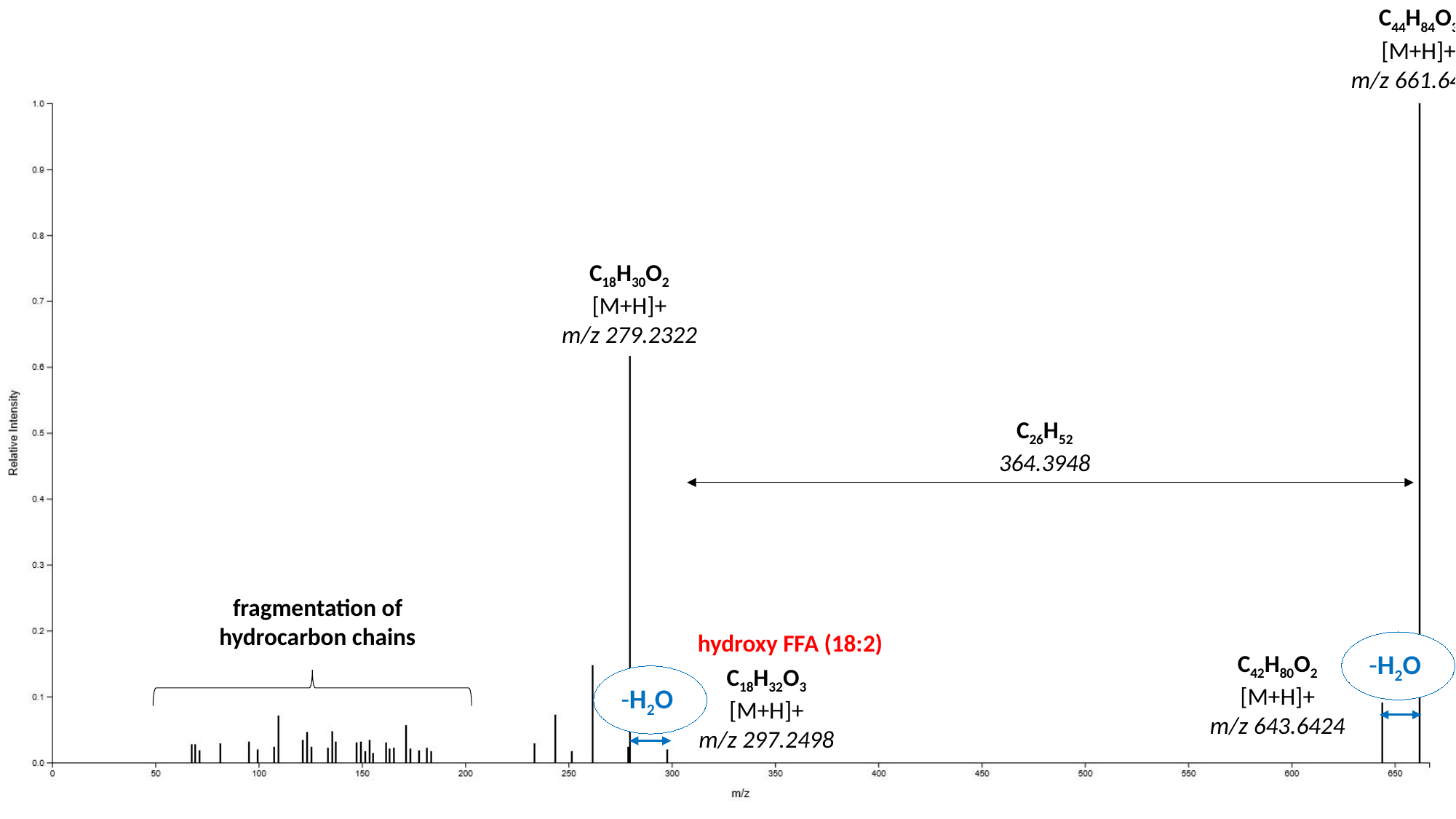

hydroxy wax ester (26:0/18:2-O)
C44H84O3
[M+H]+
m/z 661.6446
C18H30O2
[M+H]+
m/z 279.2322
C26H52
364.3948
fragmentation of hydrocarbon chains
hydroxy FFA (18:2)
-H2O
C42H80O2
[M+H]+
m/z 643.6424
C18H32O3
[M+H]+
m/z 297.2498
-H2O

## Slide 5
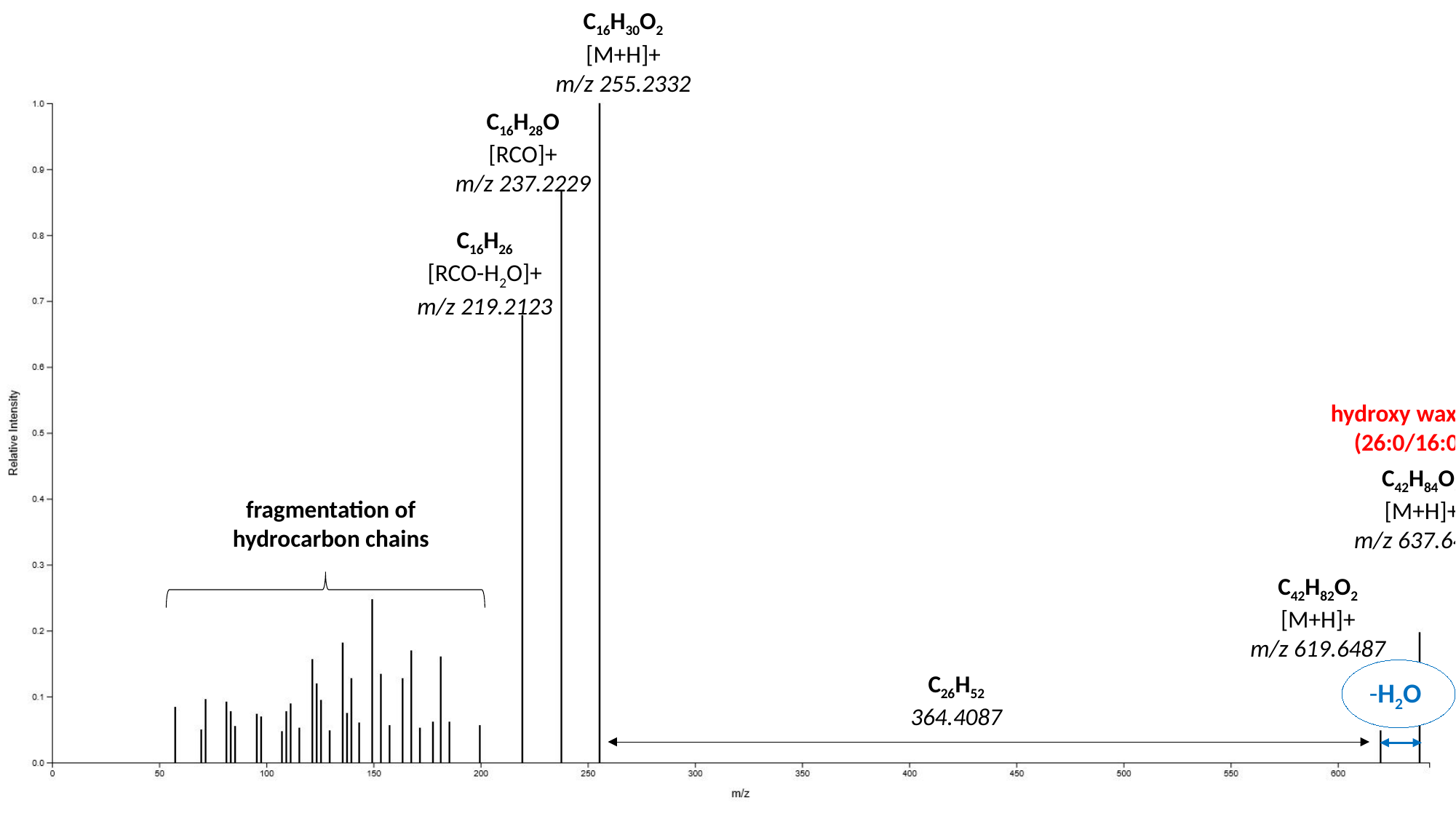

C16H30O2
[M+H]+
m/z 255.2332
C16H28O
[RCO]+
m/z 237.2229
C16H26
[RCO-H2O]+
m/z 219.2123
hydroxy wax ester (26:0/16:0-O)
C42H84O3
[M+H]+
m/z 637.6466
fragmentation of hydrocarbon chains
C42H82O2
[M+H]+
m/z 619.6487
-H2O
C26H52
364.4087

## Slide 6
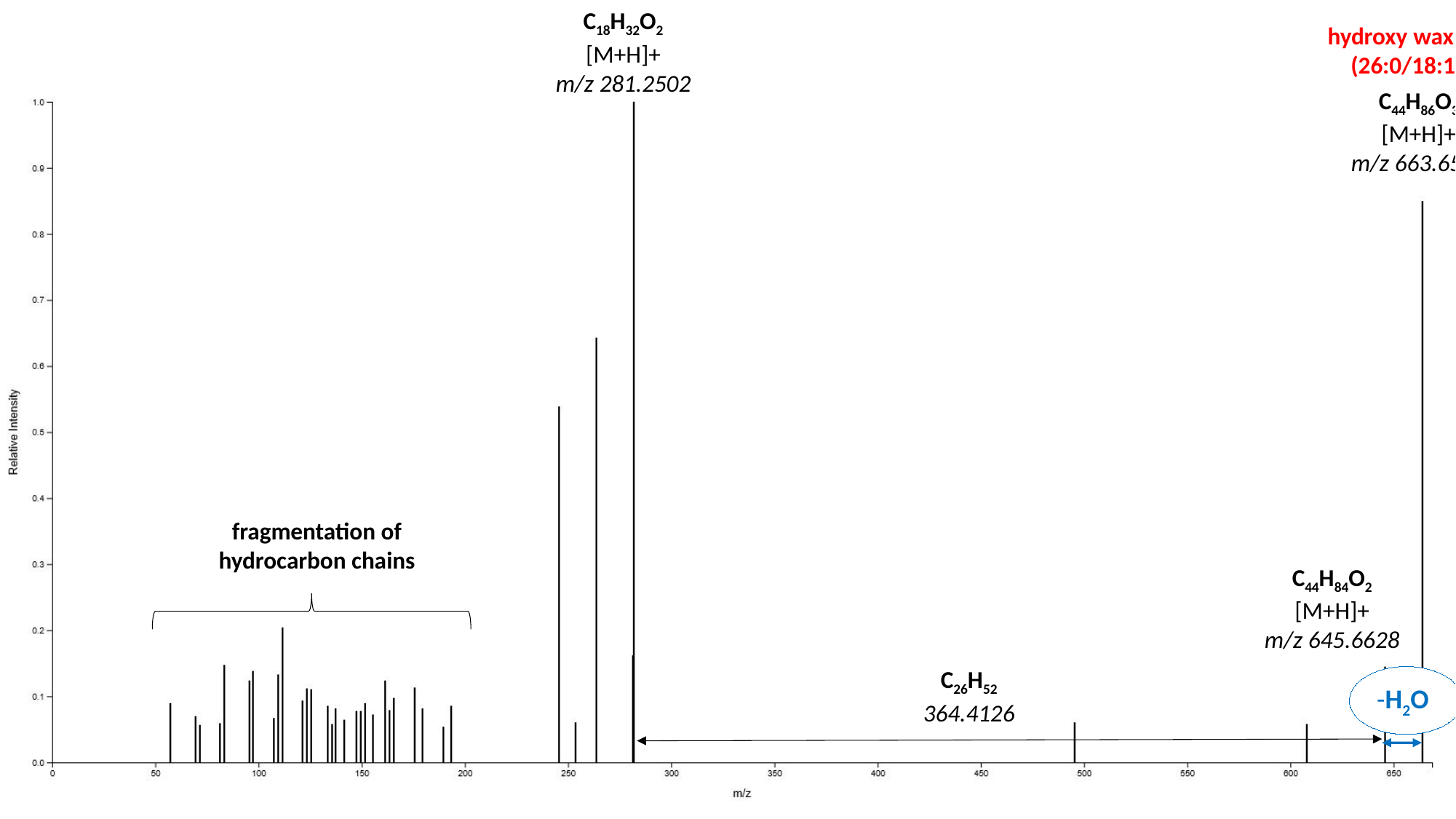

C18H32O2
[M+H]+
m/z 281.2502
hydroxy wax ester (26:0/18:1-O)
C44H86O3
[M+H]+
m/z 663.6599
fragmentation of hydrocarbon chains
C44H84O2
[M+H]+
m/z 645.6628
C26H52
364.4126
-H2O

## Slide 7
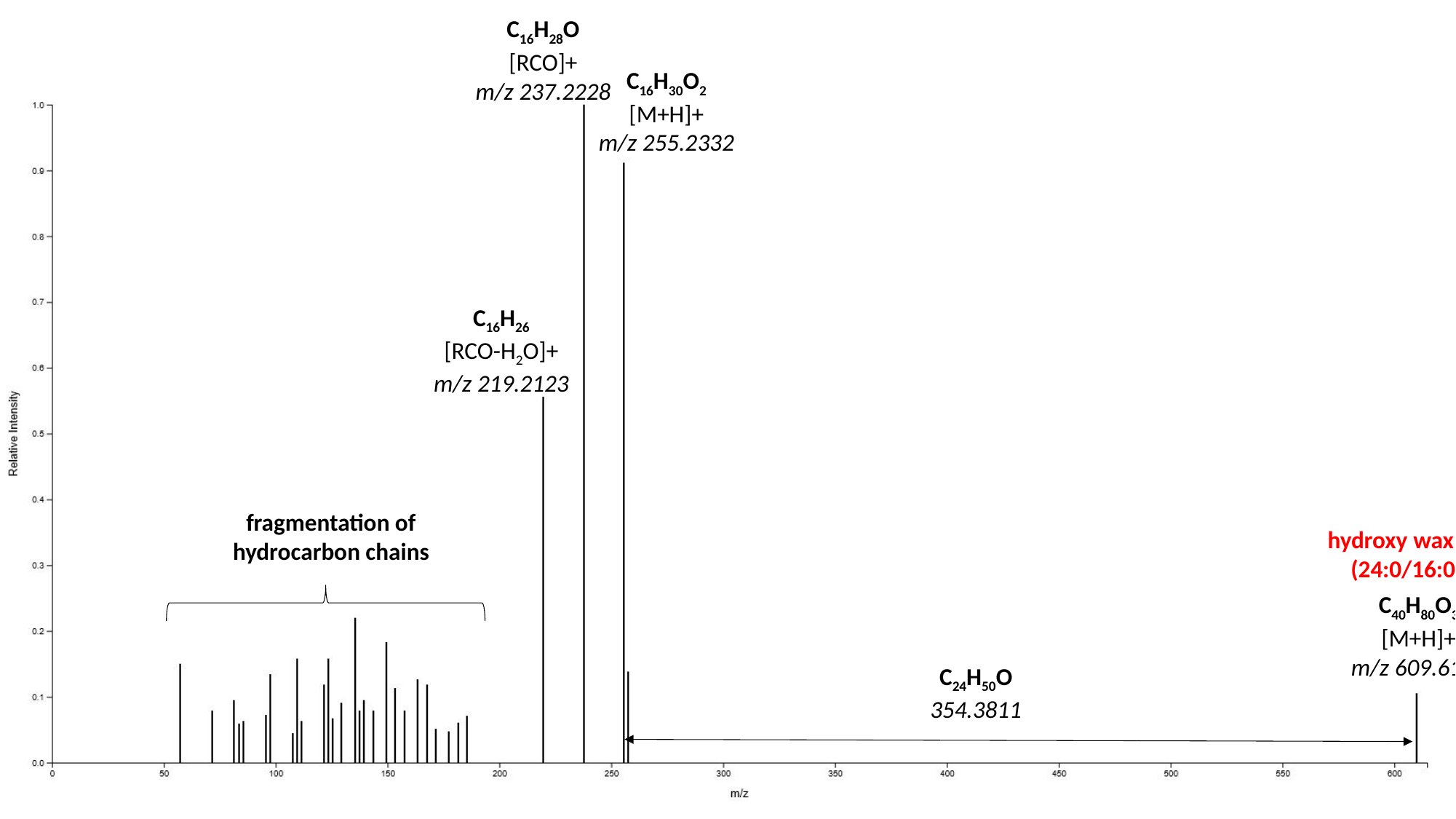

C16H28O
[RCO]+
m/z 237.2228
C16H30O2
[M+H]+
m/z 255.2332
C16H26
[RCO-H2O]+
m/z 219.2123
fragmentation of hydrocarbon chains
hydroxy wax ester (24:0/16:0-O)
C40H80O3
[M+H]+
m/z 609.6143
C24H50O
354.3811

## Slide 8
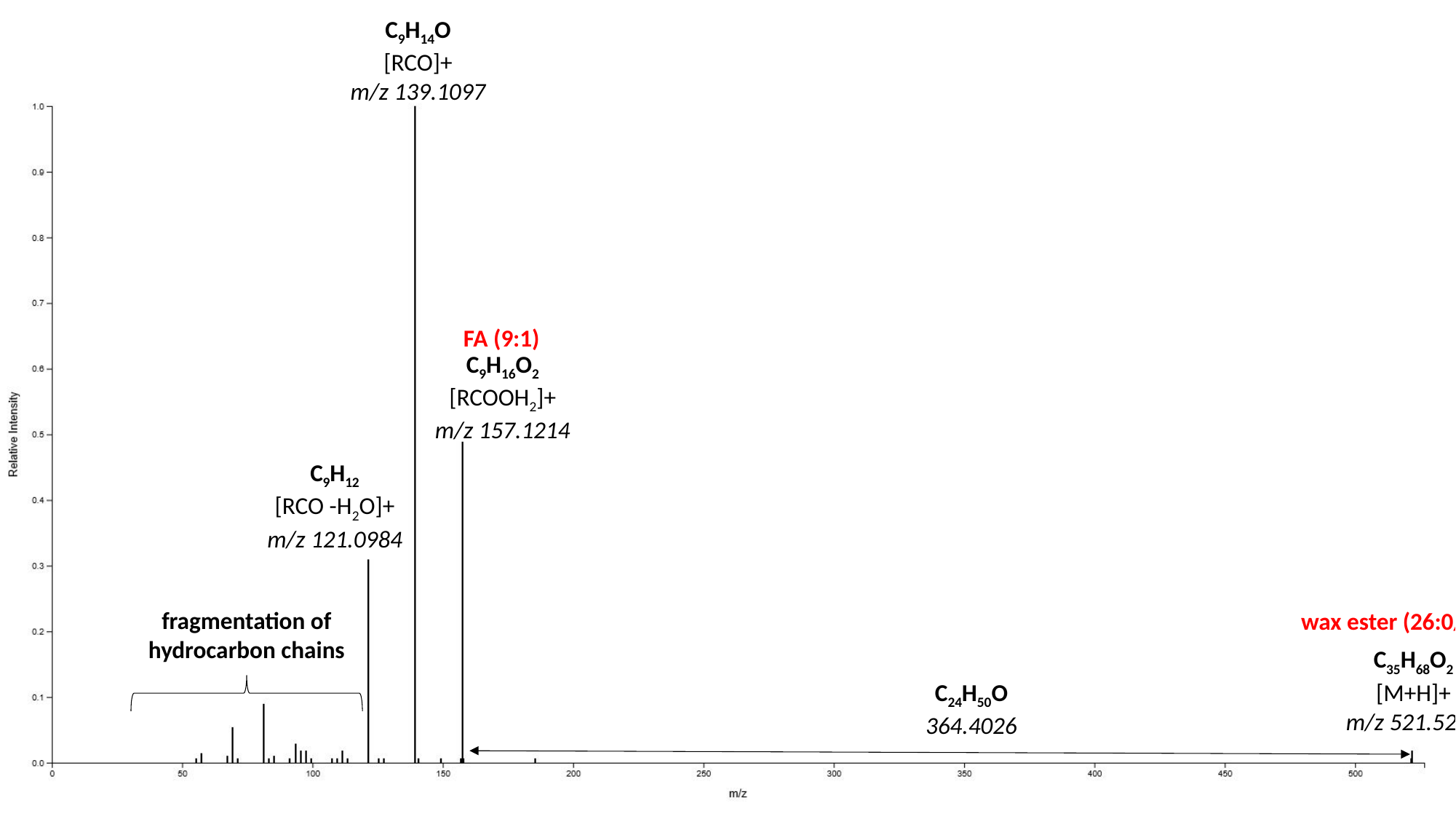

C9H14O
[RCO]+
m/z 139.1097
FA (9:1)
C9H16O2
[RCOOH2]+
m/z 157.1214
C9H12
[RCO -H2O]+
m/z 121.0984
fragmentation of hydrocarbon chains
wax ester (26:0/9:1)
C35H68O2
[M+H]+
m/z 521.5240
C24H50O
364.4026

## Slide 9
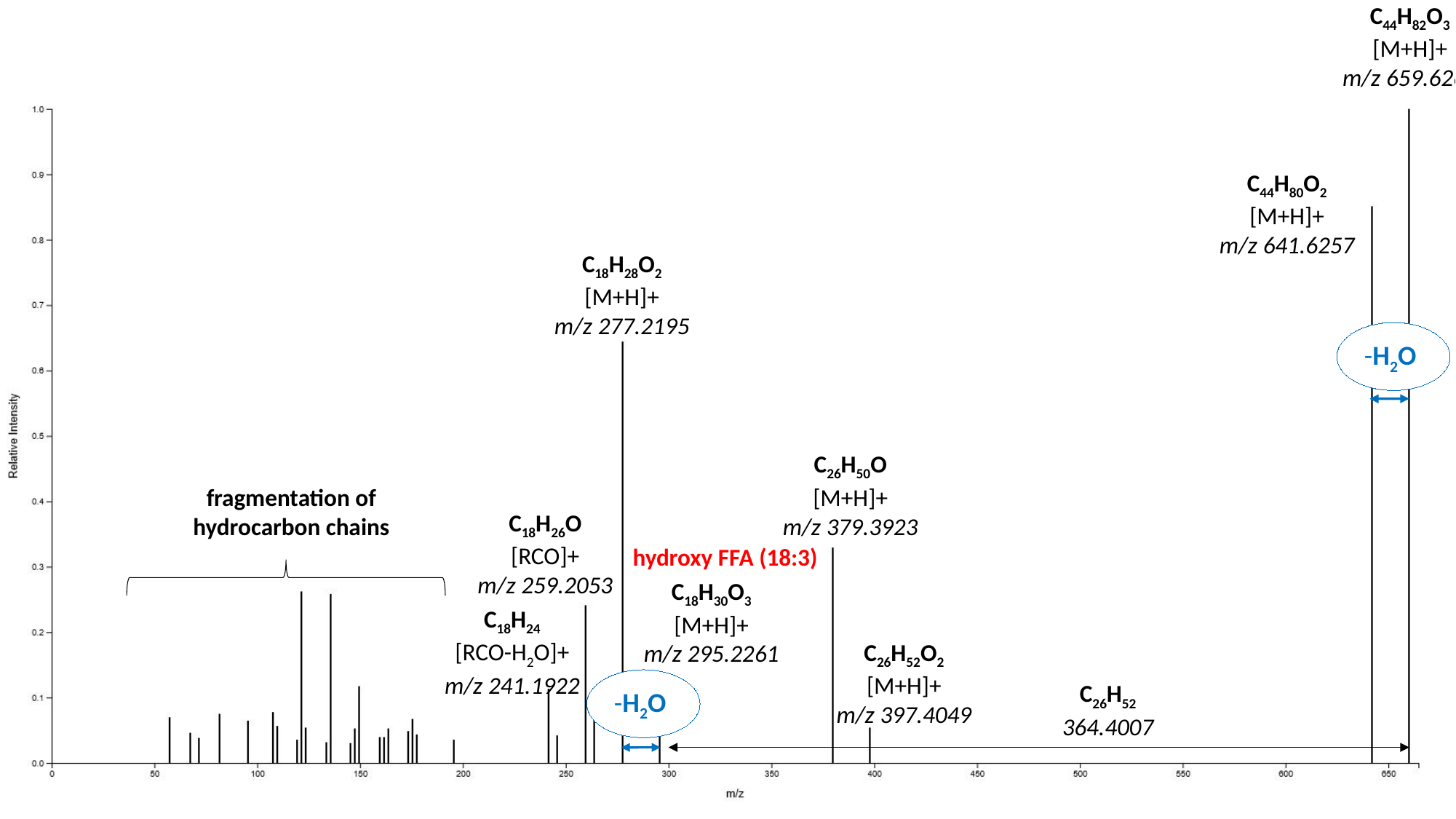

hydroxy wax ester (26:0/18:3-O)
C44H82O3
[M+H]+
m/z 659.6268
C44H80O2
[M+H]+
m/z 641.6257
C18H28O2
[M+H]+
m/z 277.2195
-H2O
C26H50O
[M+H]+
m/z 379.3923
fragmentation of hydrocarbon chains
C18H26O
[RCO]+
m/z 259.2053
hydroxy FFA (18:3)
C18H30O3
[M+H]+
m/z 295.2261
C18H24
[RCO-H2O]+
m/z 241.1922
C26H52O2
[M+H]+
m/z 397.4049
-H2O
C26H52
364.4007

## Slide 10
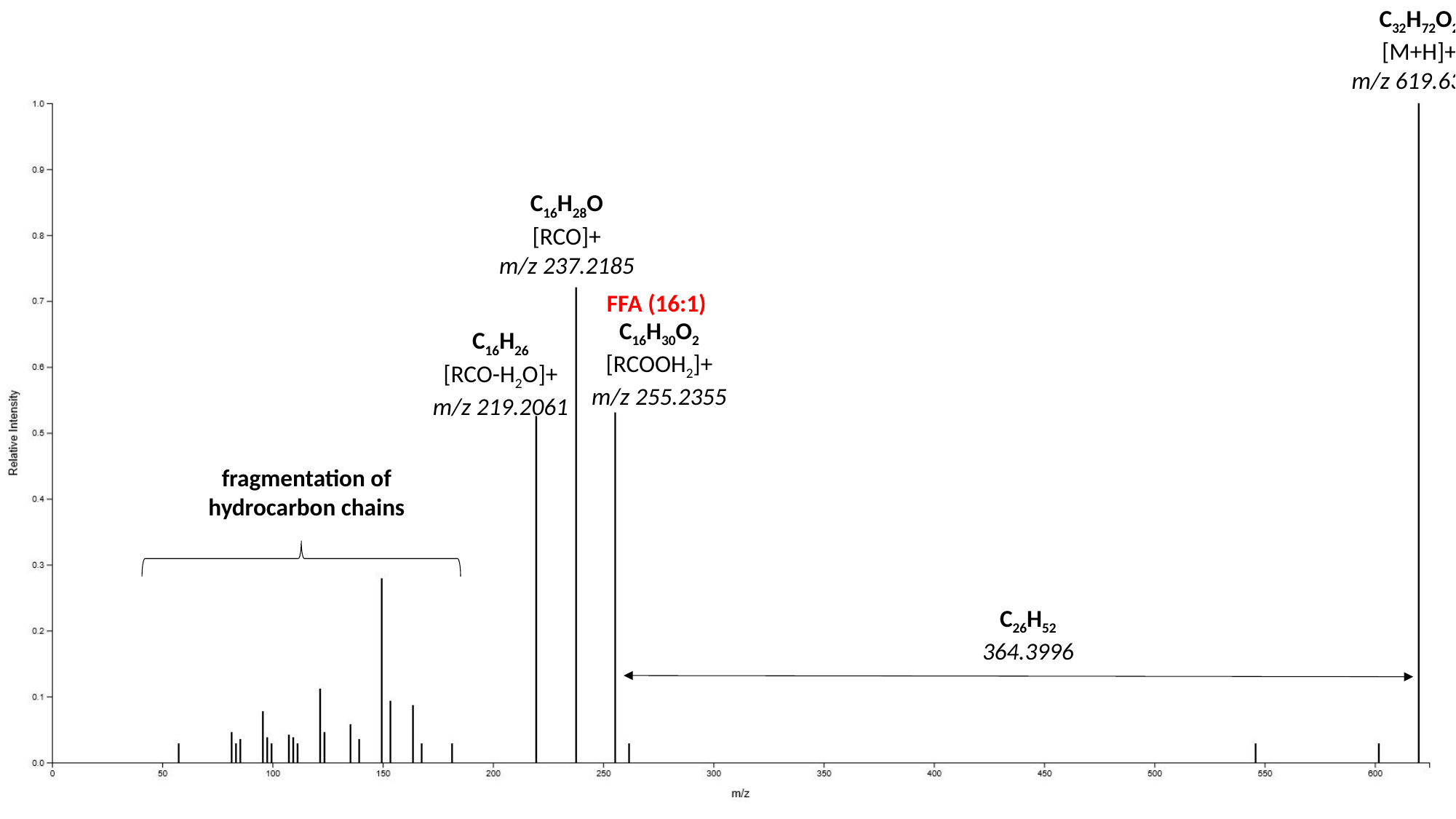

wax ester (26:0/16:1)
C32H72O2
[M+H]+
m/z 619.6351
C16H28O
[RCO]+
m/z 237.2185
FFA (16:1)
C16H30O2
[RCOOH2]+
m/z 255.2355
C16H26
[RCO-H2O]+
m/z 219.2061
fragmentation of hydrocarbon chains
C26H52
364.3996

## Slide 11
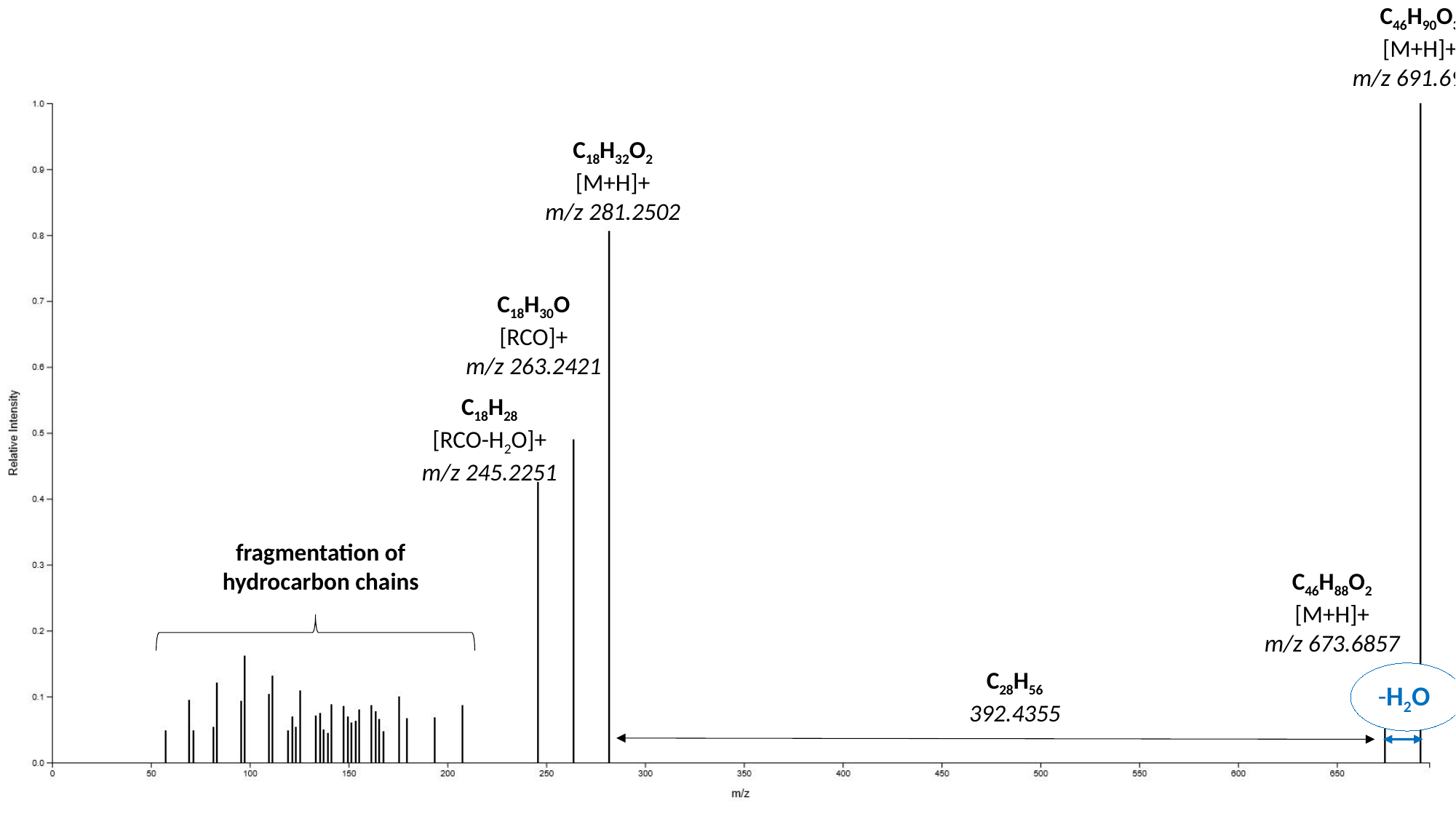

hydroxy wax ester (28:0/18:1-O)
C46H90O3
[M+H]+
m/z 691.6917
C18H32O2
[M+H]+
m/z 281.2502
C18H30O
[RCO]+
m/z 263.2421
C18H28
[RCO-H2O]+
m/z 245.2251
fragmentation of hydrocarbon chains
C46H88O2
[M+H]+
m/z 673.6857
C28H56
392.4355
-H2O

## Slide 12
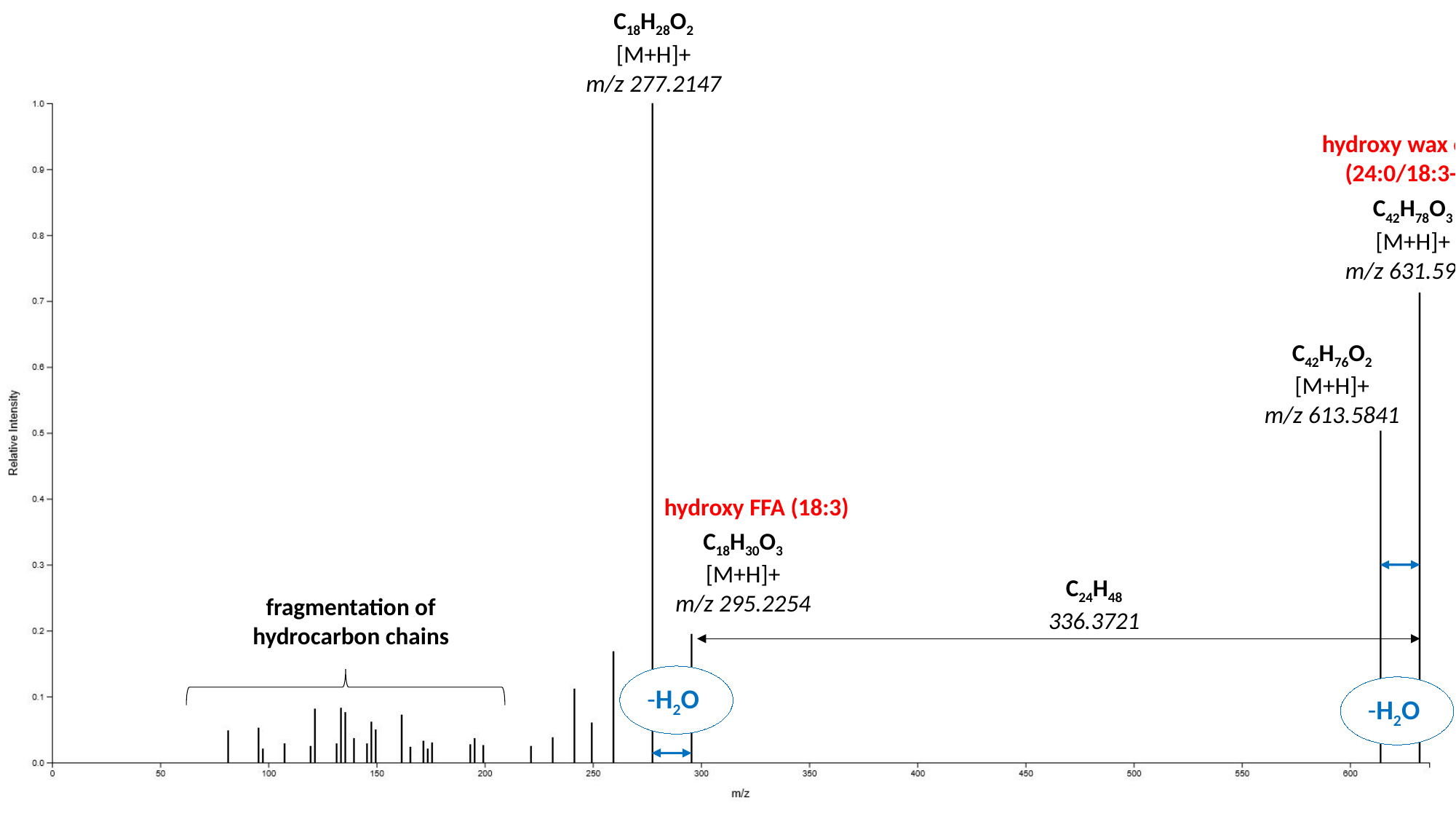

C18H28O2
[M+H]+
m/z 277.2147
hydroxy wax ester (24:0/18:3-O)
C42H78O3
[M+H]+
m/z 631.5975
C42H76O2
[M+H]+
m/z 613.5841
hydroxy FFA (18:3)
C18H30O3
[M+H]+
m/z 295.2254
C24H48
336.3721
fragmentation of hydrocarbon chains
-H2O
-H2O

## Slide 13
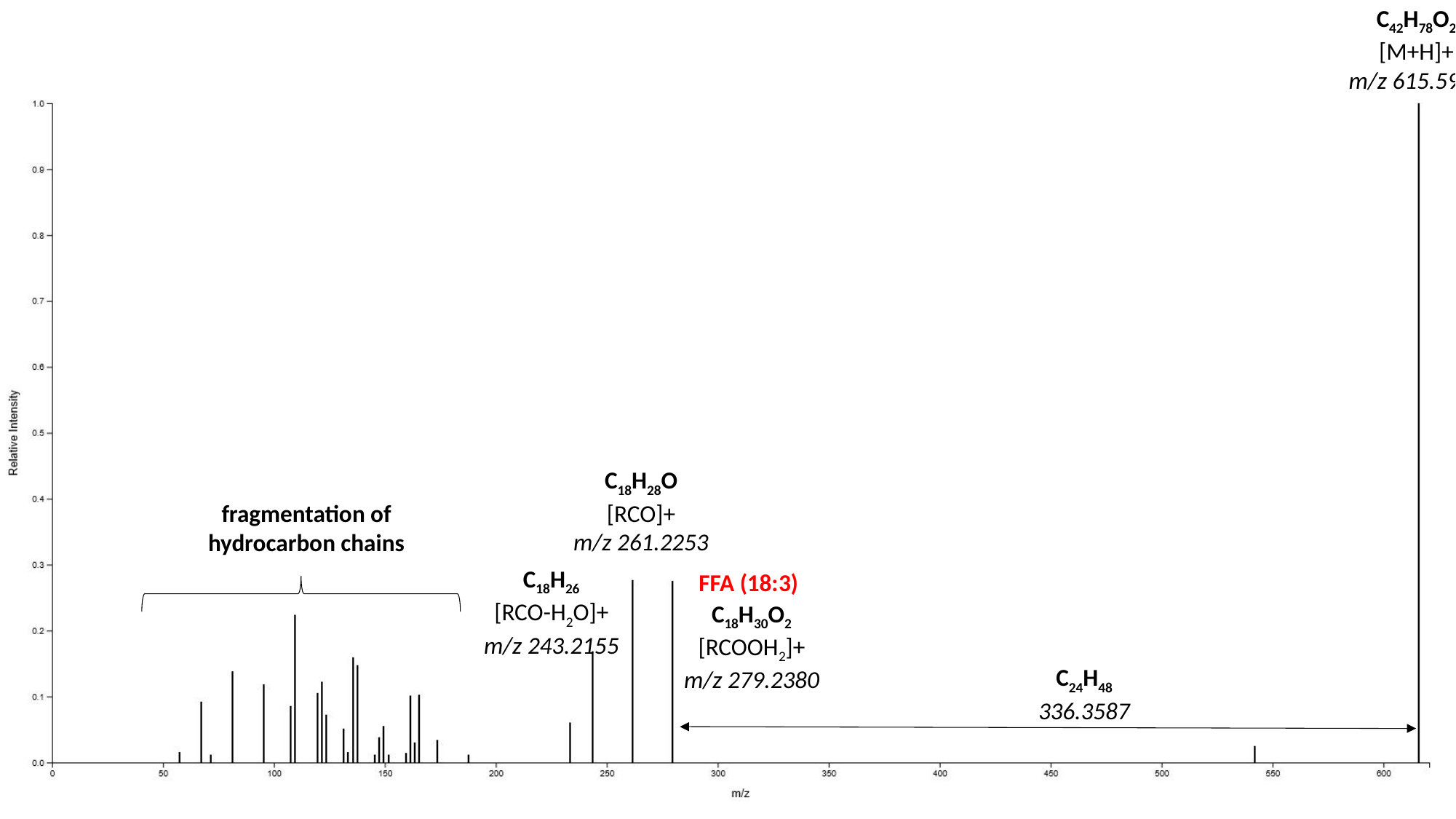

wax ester (24:0/18:3)
C42H78O2
[M+H]+
m/z 615.5967
C18H28O
[RCO]+
m/z 261.2253
fragmentation of hydrocarbon chains
C18H26
[RCO-H2O]+
m/z 243.2155
FFA (18:3)
C18H30O2
[RCOOH2]+
m/z 279.2380
C24H48
336.3587

## Slide 14
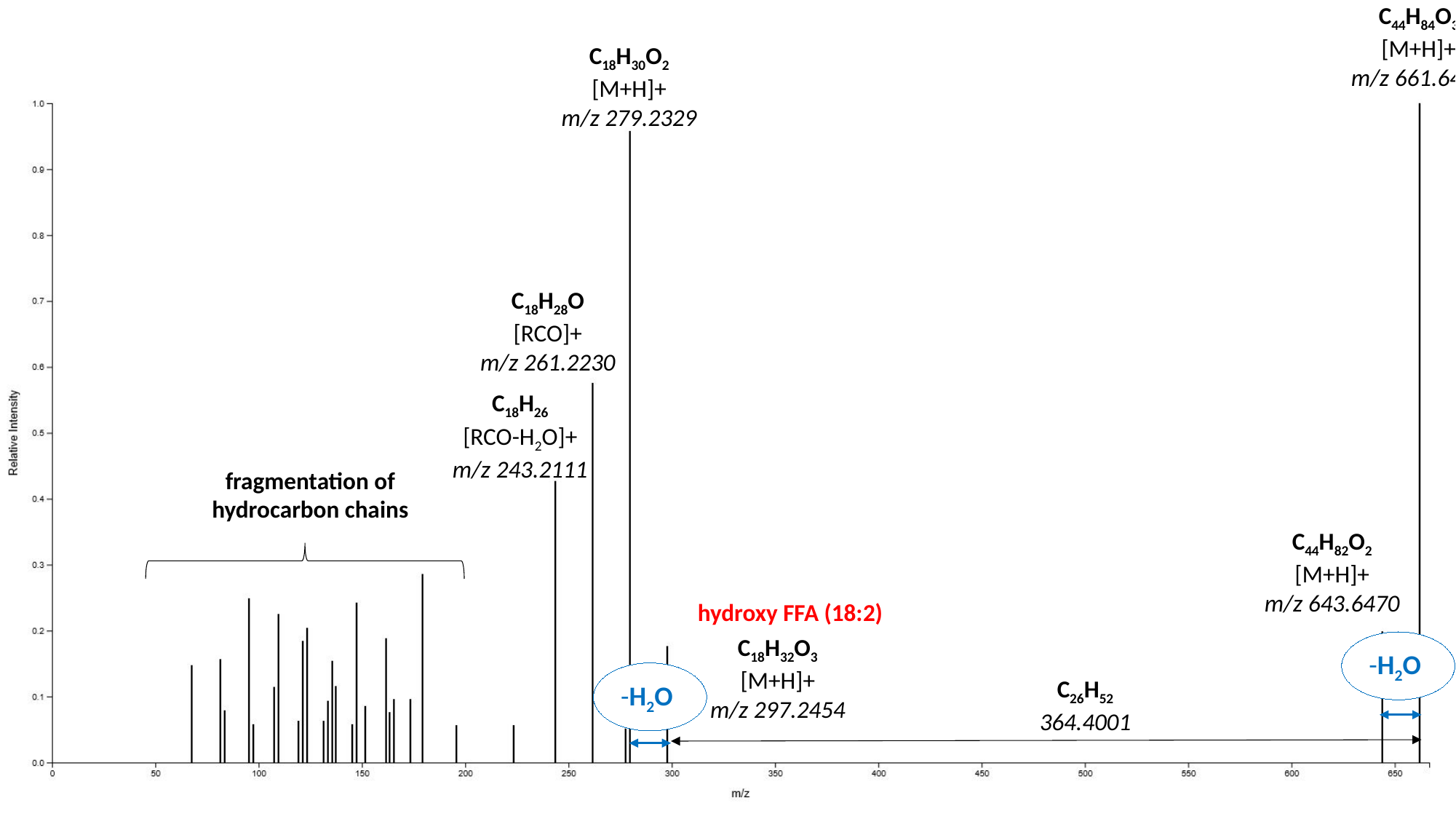

hydroxy wax ester (24:0/18:2-O)
C44H84O3
[M+H]+
m/z 661.6455
C18H30O2
[M+H]+
m/z 279.2329
C18H28O
[RCO]+
m/z 261.2230
C18H26
[RCO-H2O]+
m/z 243.2111
fragmentation of hydrocarbon chains
C44H82O2
[M+H]+
m/z 643.6470
hydroxy FFA (18:2)
C18H32O3
[M+H]+
m/z 297.2454
-H2O
-H2O
C26H52
364.4001

## Slide 15
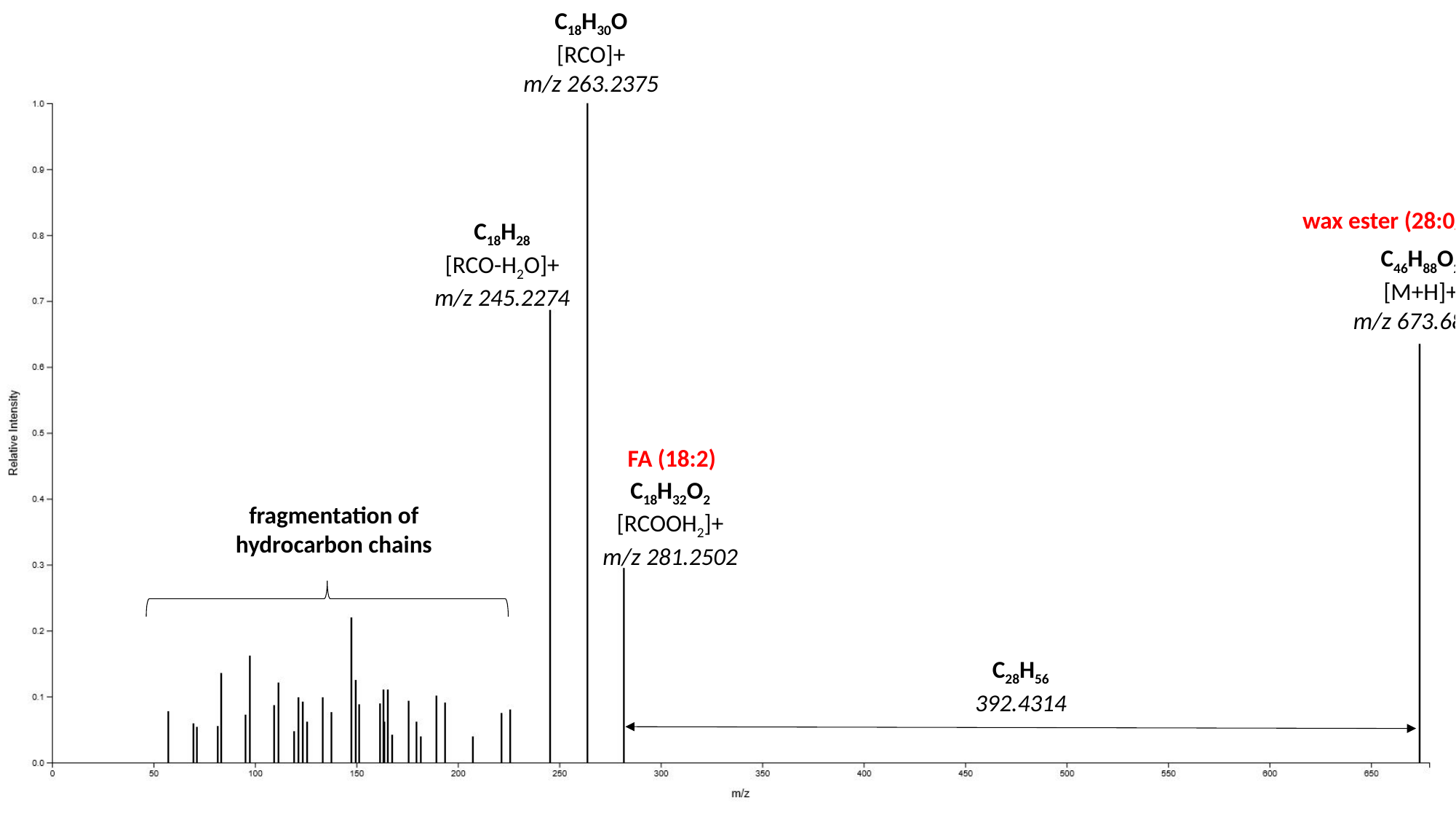

C18H30O
[RCO]+
m/z 263.2375
wax ester (28:0/18:2)
C18H28
[RCO-H2O]+
m/z 245.2274
C46H88O2
[M+H]+
m/z 673.6816
FA (18:2)
C18H32O2
[RCOOH2]+
m/z 281.2502
fragmentation of hydrocarbon chains
C28H56
392.4314

## Slide 16
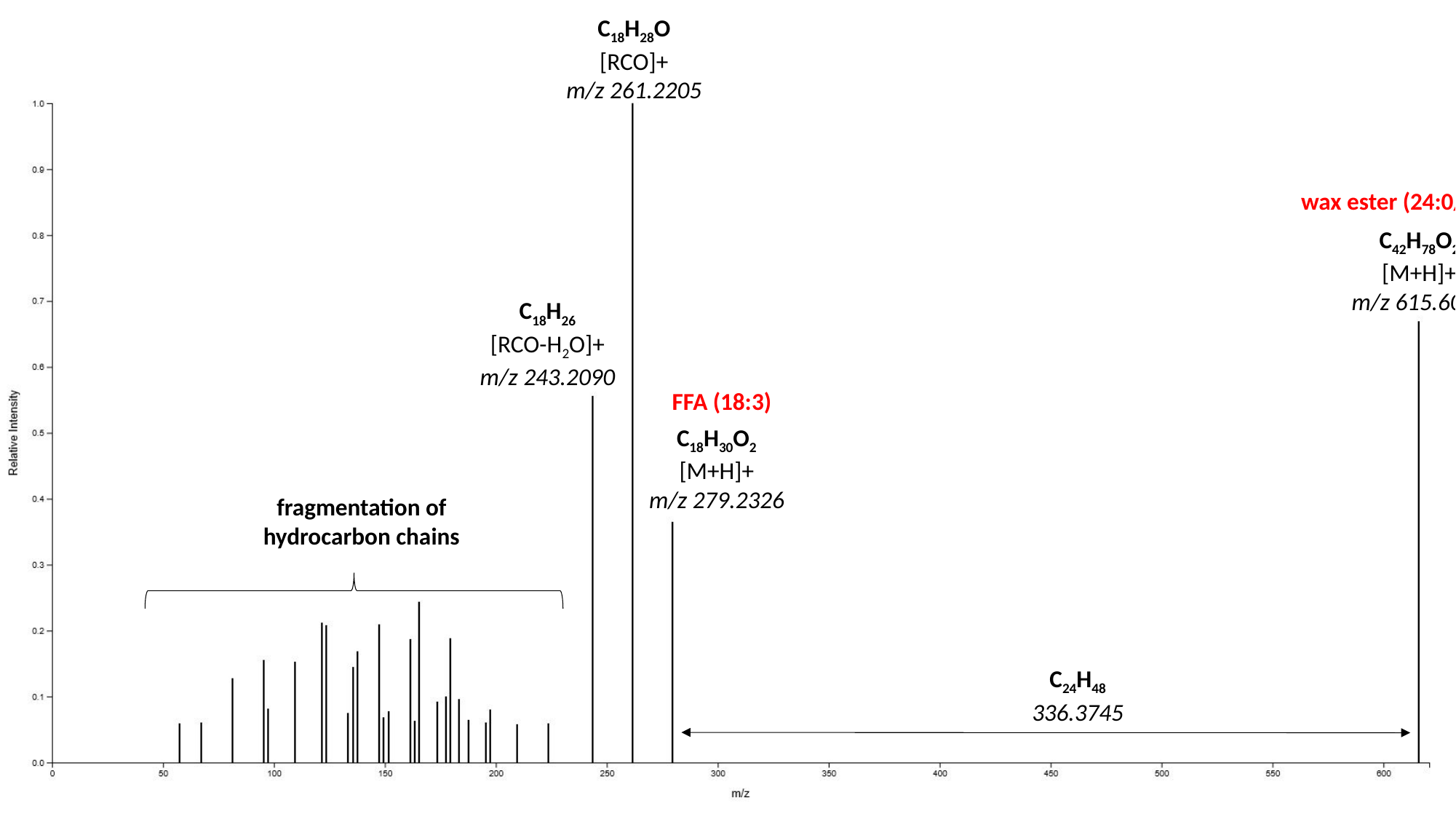

C18H28O
[RCO]+
m/z 261.2205
wax ester (24:0/18:3)
C42H78O2
[M+H]+
m/z 615.6071
C18H26
[RCO-H2O]+
m/z 243.2090
FFA (18:3)
C18H30O2
[M+H]+
m/z 279.2326
fragmentation of hydrocarbon chains
C24H48
336.3745

## Slide 17
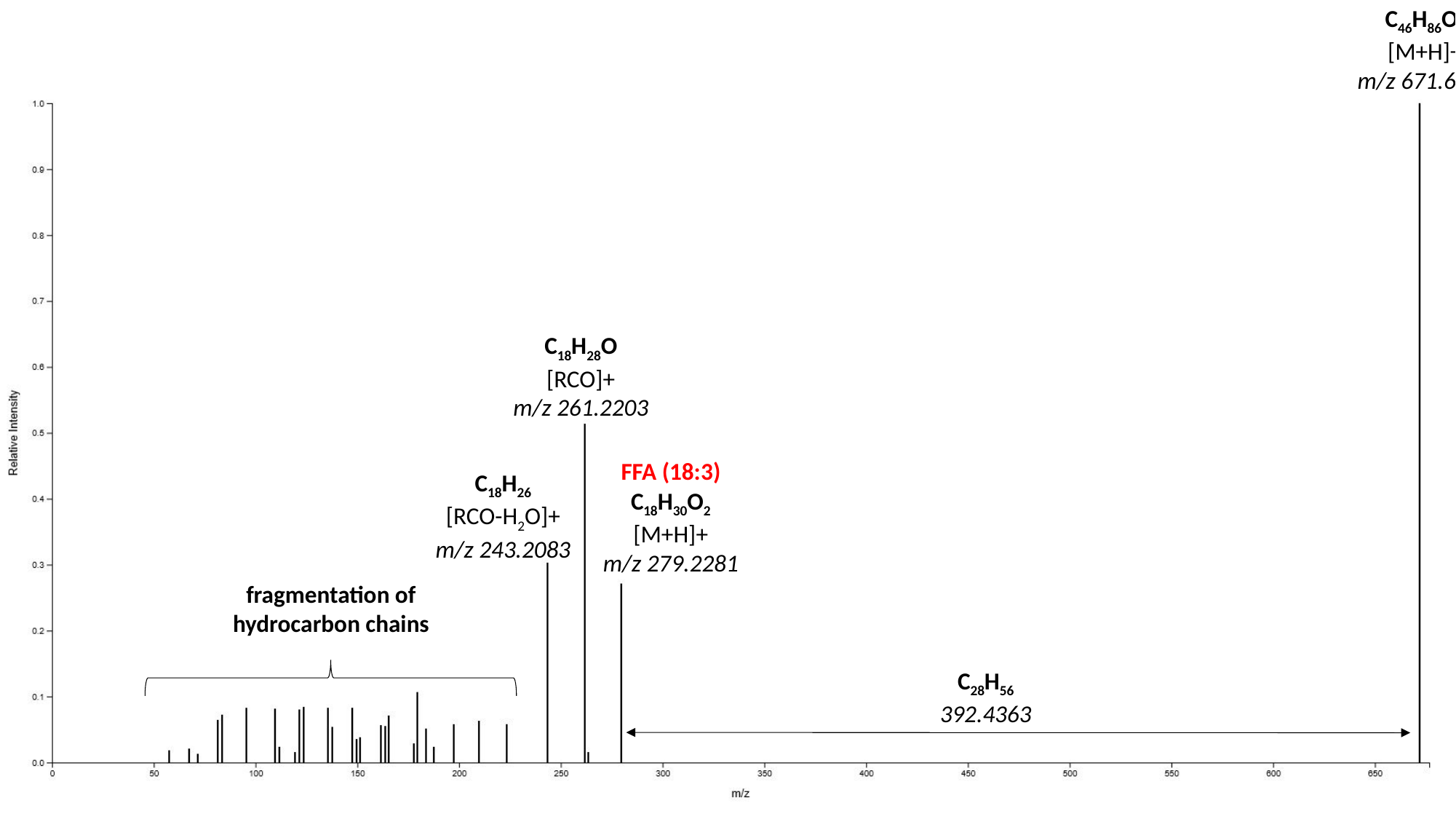

wax ester (28:0/18:3)
C46H86O2
[M+H]+
m/z 671.6644
C18H28O
[RCO]+
m/z 261.2203
FFA (18:3)
C18H26
[RCO-H2O]+
m/z 243.2083
C18H30O2
[M+H]+
m/z 279.2281
fragmentation of hydrocarbon chains
C28H56
392.4363

## Slide 18
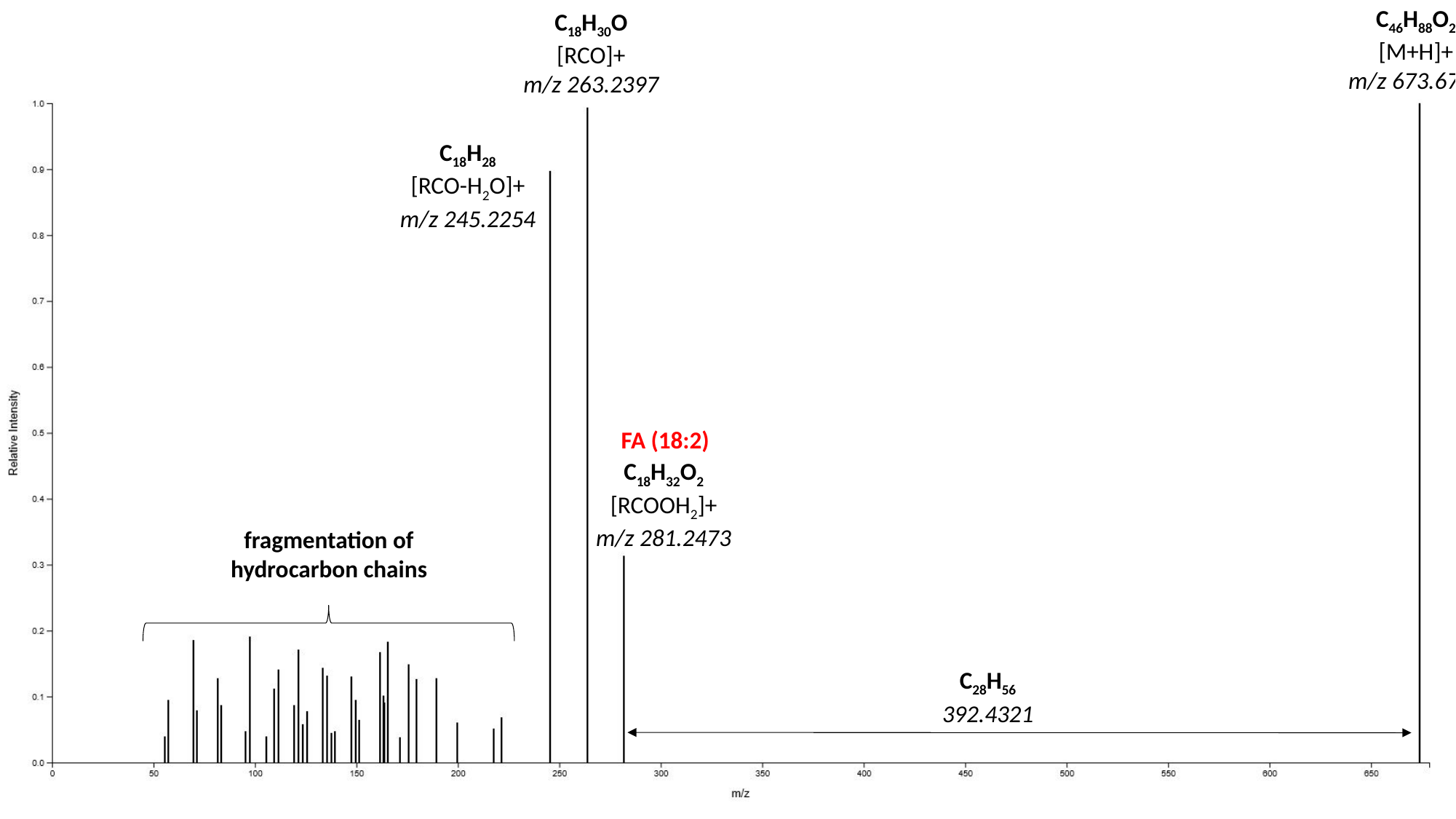

wax ester (28:0/18:2)
C46H88O2
[M+H]+
m/z 673.6794
C18H30O
[RCO]+
m/z 263.2397
C18H28
[RCO-H2O]+
m/z 245.2254
FA (18:2)
C18H32O2
[RCOOH2]+
m/z 281.2473
fragmentation of hydrocarbon chains
C28H56
392.4321

## Slide 19
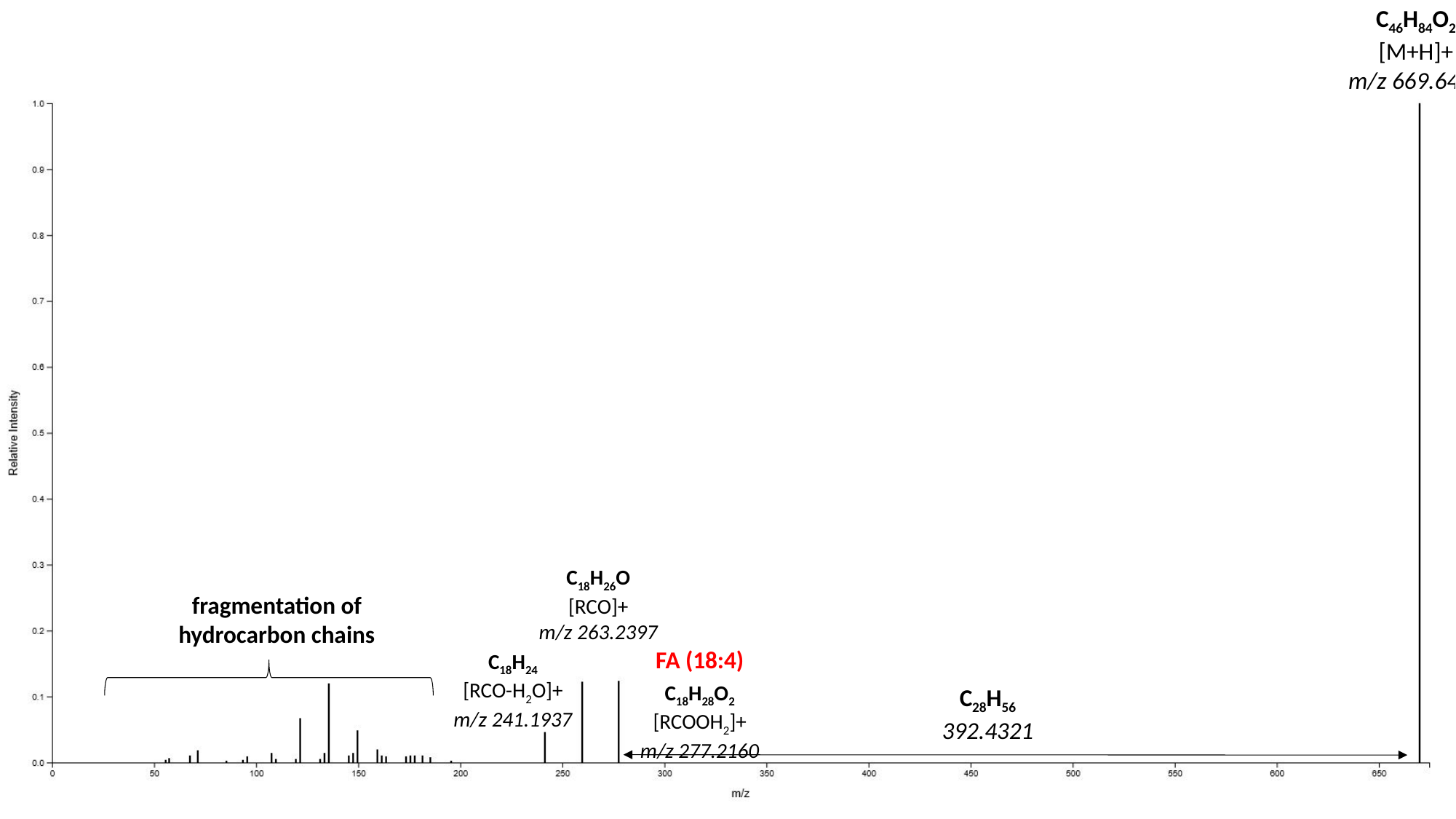

wax ester (28:0/18:4)
C46H84O2
[M+H]+
m/z 669.6464
C18H26O
[RCO]+
m/z 263.2397
fragmentation of hydrocarbon chains
FA (18:4)
C18H24
[RCO-H2O]+
m/z 241.1937
C18H28O2
[RCOOH2]+
m/z 277.2160
C28H56
392.4321

## Slide 20
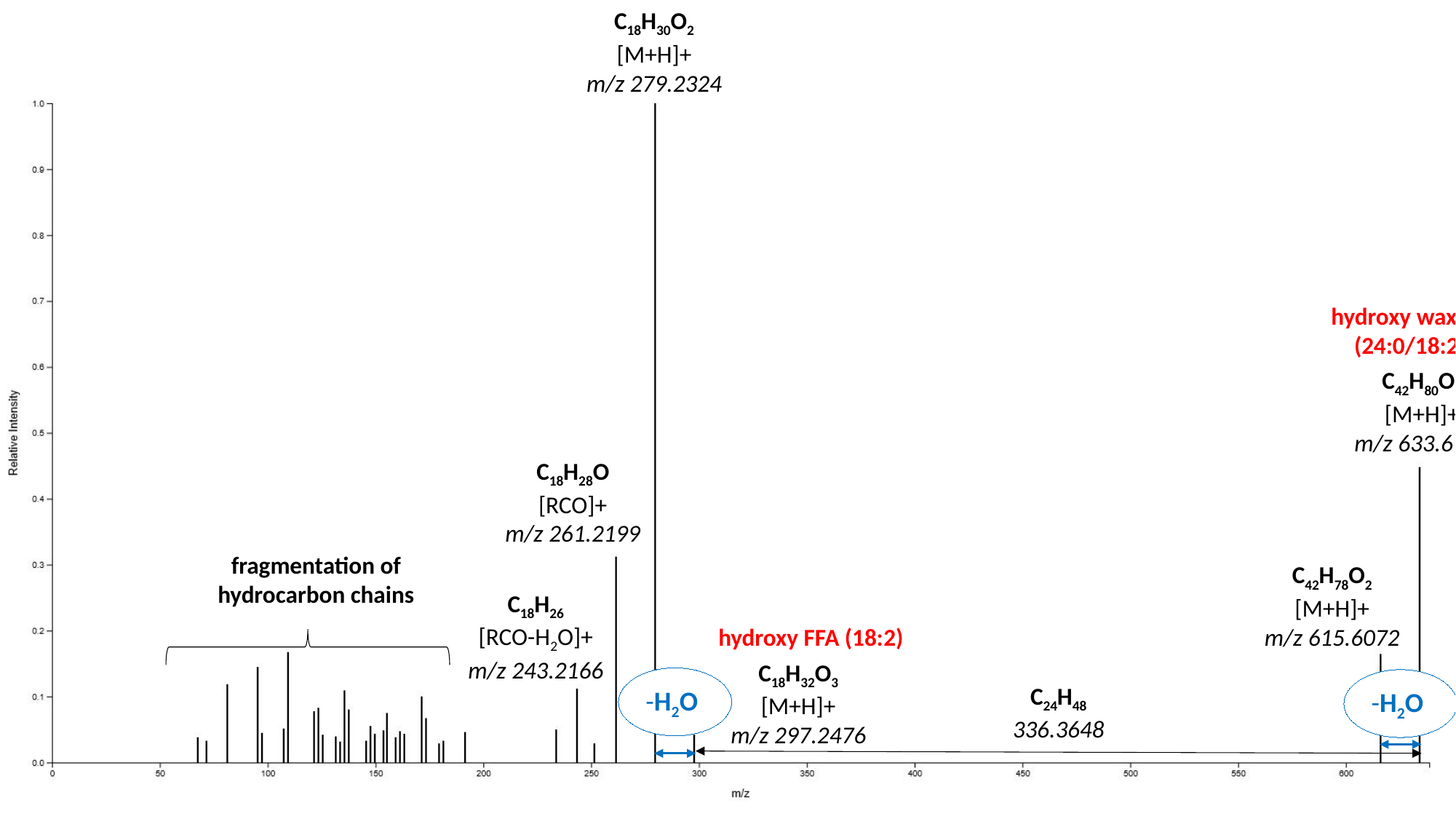

C18H30O2
[M+H]+
m/z 279.2324
hydroxy wax ester (24:0/18:2-O)
C42H80O3
[M+H]+
m/z 633.6124
C18H28O
[RCO]+
m/z 261.2199
fragmentation of hydrocarbon chains
C42H78O2
[M+H]+
m/z 615.6072
C18H26
[RCO-H2O]+
m/z 243.2166
hydroxy FFA (18:2)
C18H32O3
[M+H]+
m/z 297.2476
-H2O
-H2O
C24H48
336.3648

## Slide 21
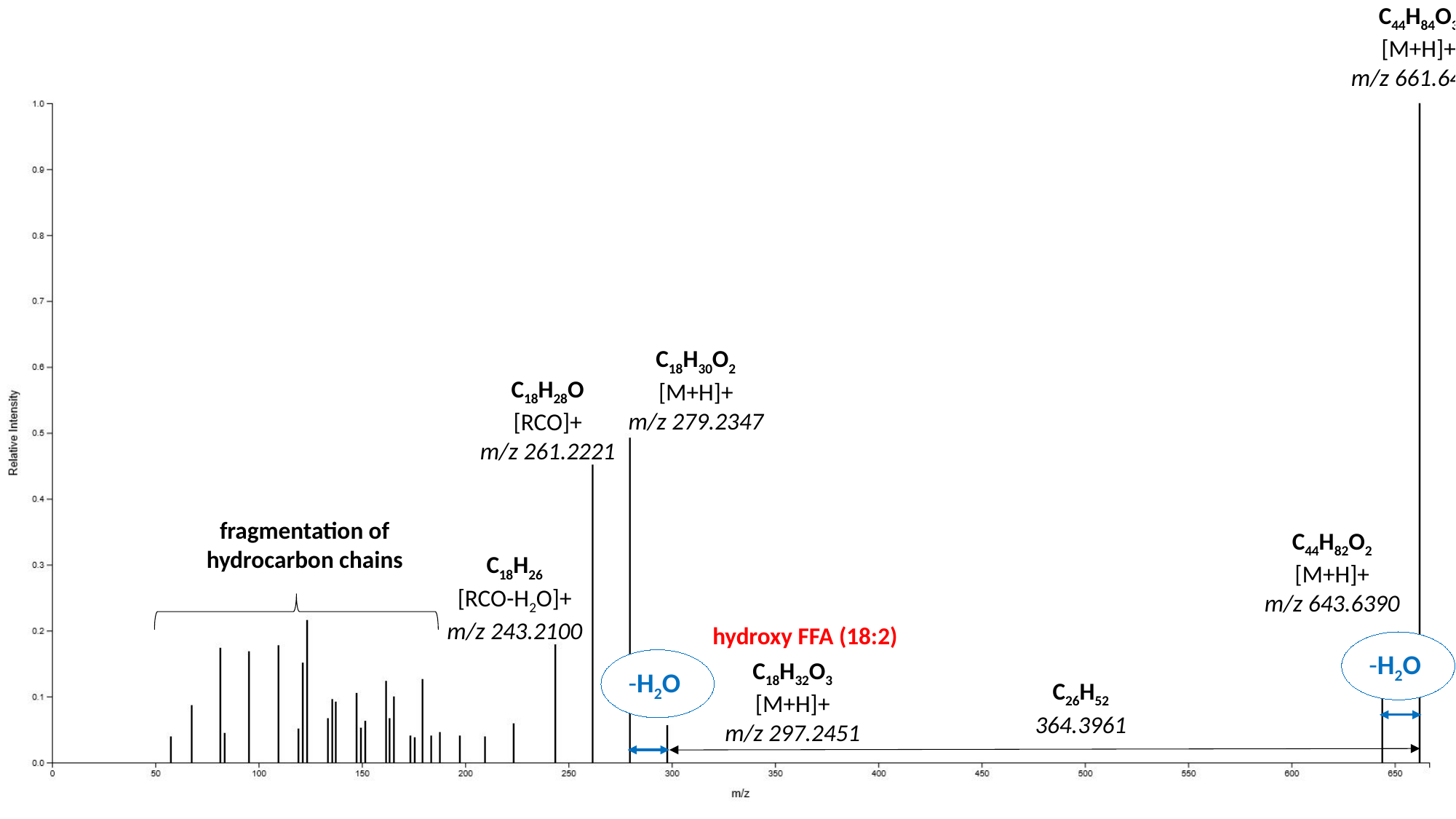

hydroxy wax ester (24:0/18:2-O)
C44H84O3
[M+H]+
m/z 661.6455
C18H30O2
[M+H]+
m/z 279.2347
C18H28O
[RCO]+
m/z 261.2221
fragmentation of hydrocarbon chains
C44H82O2
[M+H]+
m/z 643.6390
C18H26
[RCO-H2O]+
m/z 243.2100
hydroxy FFA (18:2)
-H2O
-H2O
C18H32O3
[M+H]+
m/z 297.2451
C26H52
364.3961

## Slide 22
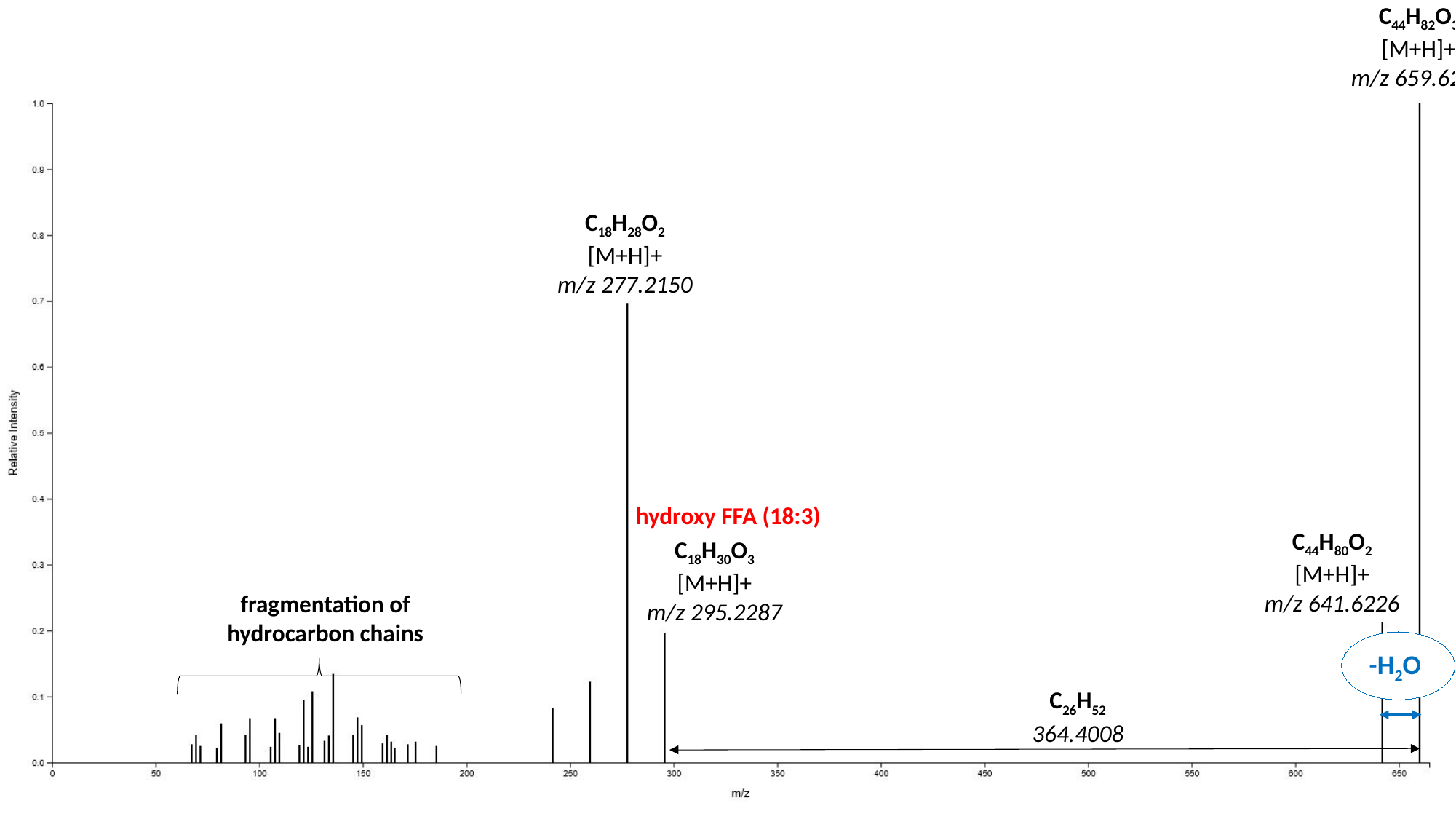

hydroxy wax ester (26:0/18:3-O)
C44H82O3
[M+H]+
m/z 659.6295
C18H28O2
[M+H]+
m/z 277.2150
hydroxy FFA (18:3)
C44H80O2
[M+H]+
m/z 641.6226
C18H30O3
[M+H]+
m/z 295.2287
fragmentation of hydrocarbon chains
-H2O
C26H52
364.4008

## Slide 23
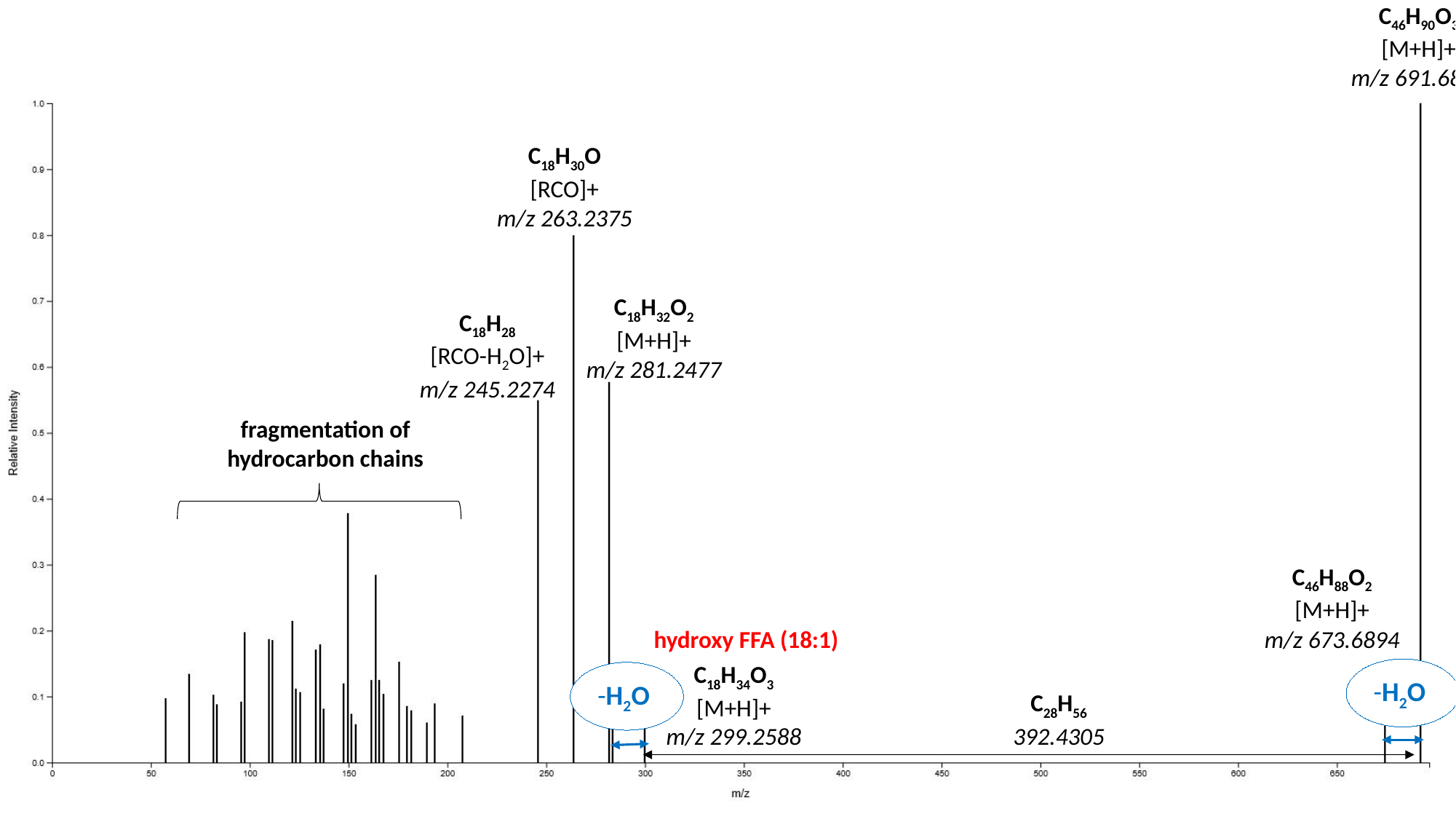

hydroxy wax ester (28:0/18:1-O)
C46H90O3
[M+H]+
m/z 691.6893
C18H30O
[RCO]+
m/z 263.2375
C18H32O2
[M+H]+
m/z 281.2477
C18H28
[RCO-H2O]+
m/z 245.2274
fragmentation of hydrocarbon chains
C46H88O2
[M+H]+
m/z 673.6894
hydroxy FFA (18:1)
C18H34O3
[M+H]+
m/z 299.2588
-H2O
-H2O
C28H56
392.4305

## Slide 24
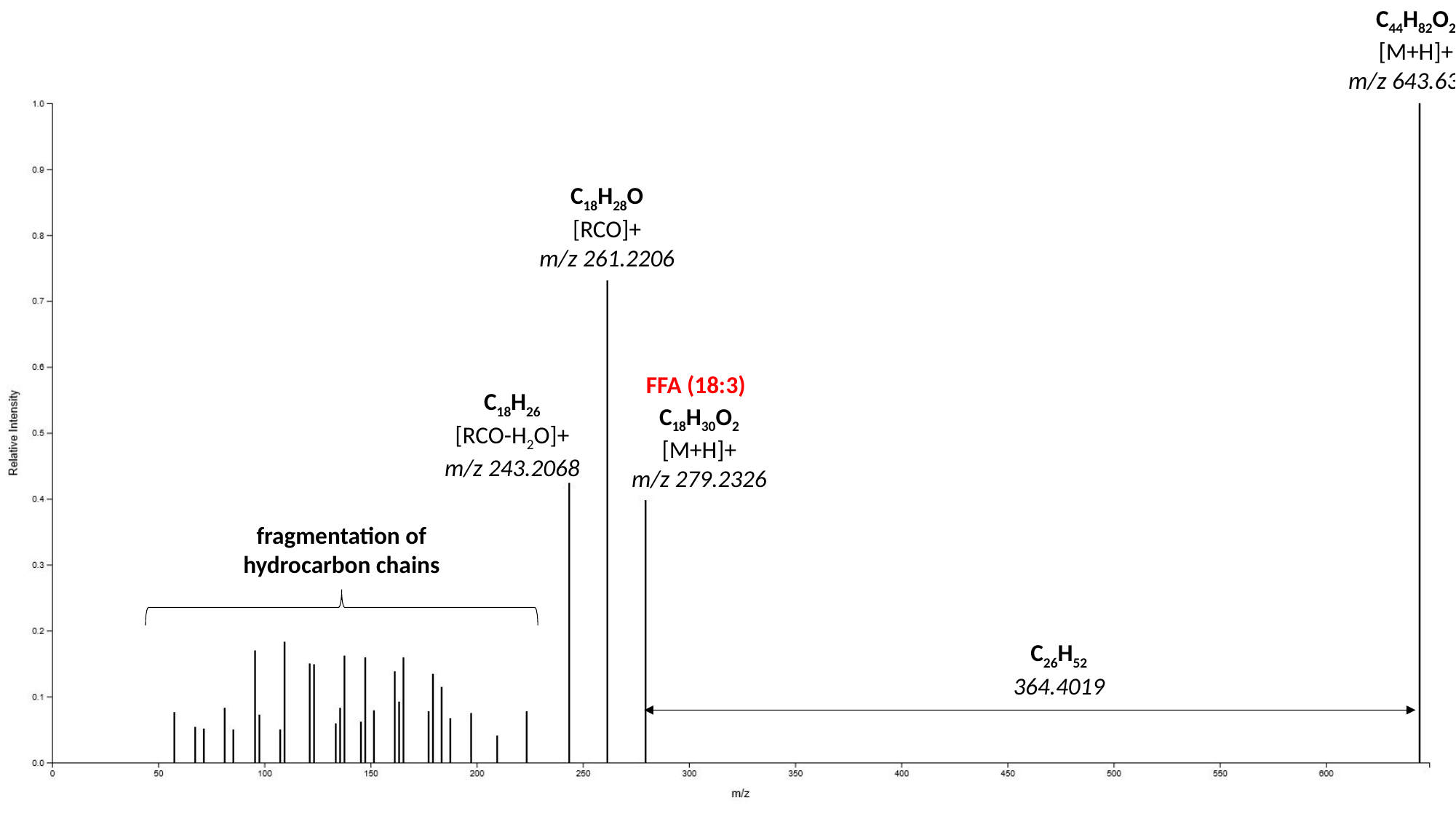

wax ester (26:0/18:3)
C44H82O2
[M+H]+
m/z 643.6345
C18H28O
[RCO]+
m/z 261.2206
FFA (18:3)
C18H26
[RCO-H2O]+
m/z 243.2068
C18H30O2
[M+H]+
m/z 279.2326
fragmentation of hydrocarbon chains
C26H52
364.4019

## Slide 25
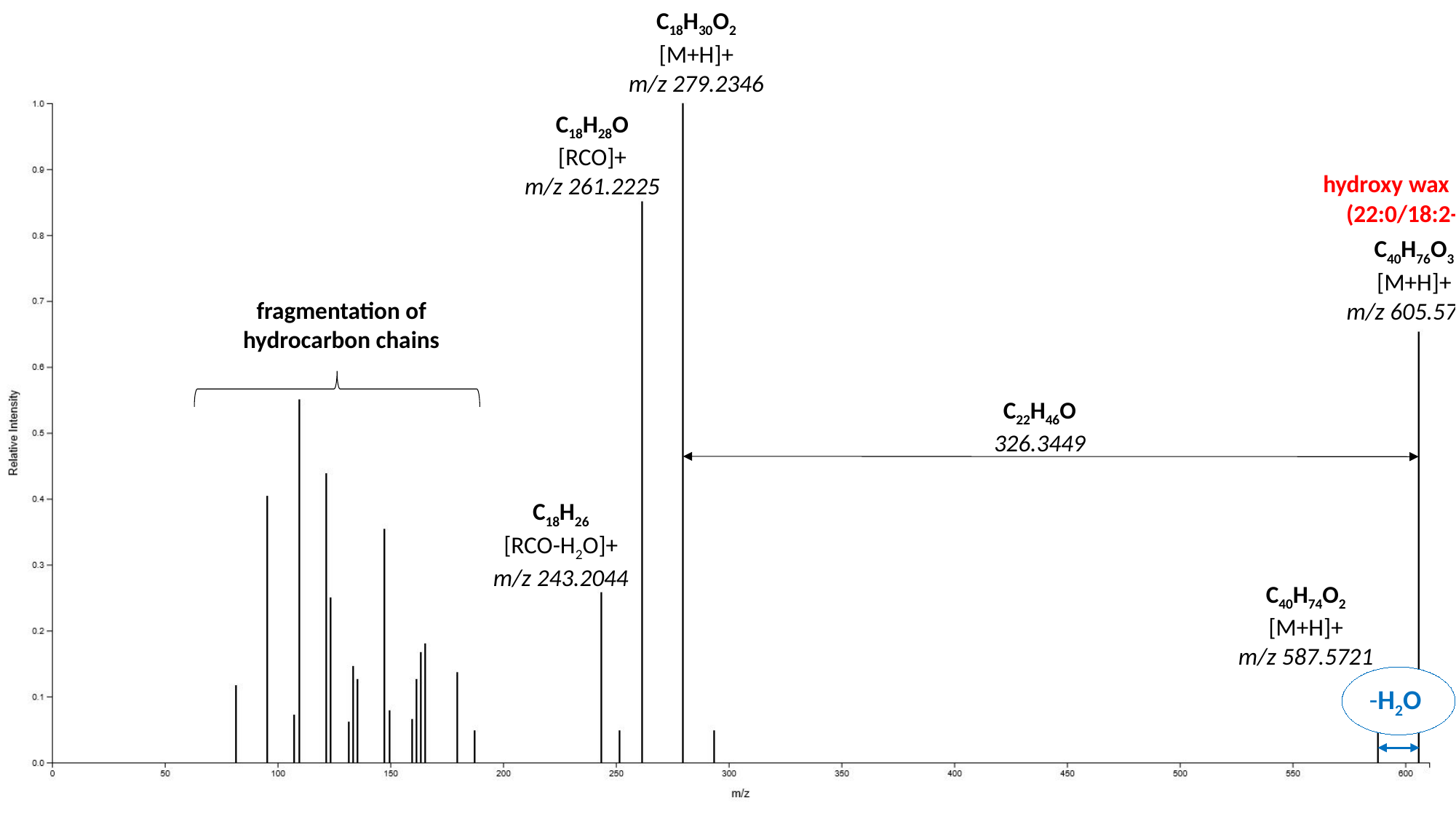

C18H30O2
[M+H]+
m/z 279.2346
C18H28O
[RCO]+
m/z 261.2225
hydroxy wax ester (22:0/18:2-O)
C40H76O3
[M+H]+
m/z 605.5795
fragmentation of hydrocarbon chains
C22H46O
326.3449
C18H26
[RCO-H2O]+
m/z 243.2044
C40H74O2
[M+H]+
m/z 587.5721
-H2O

## Slide 26
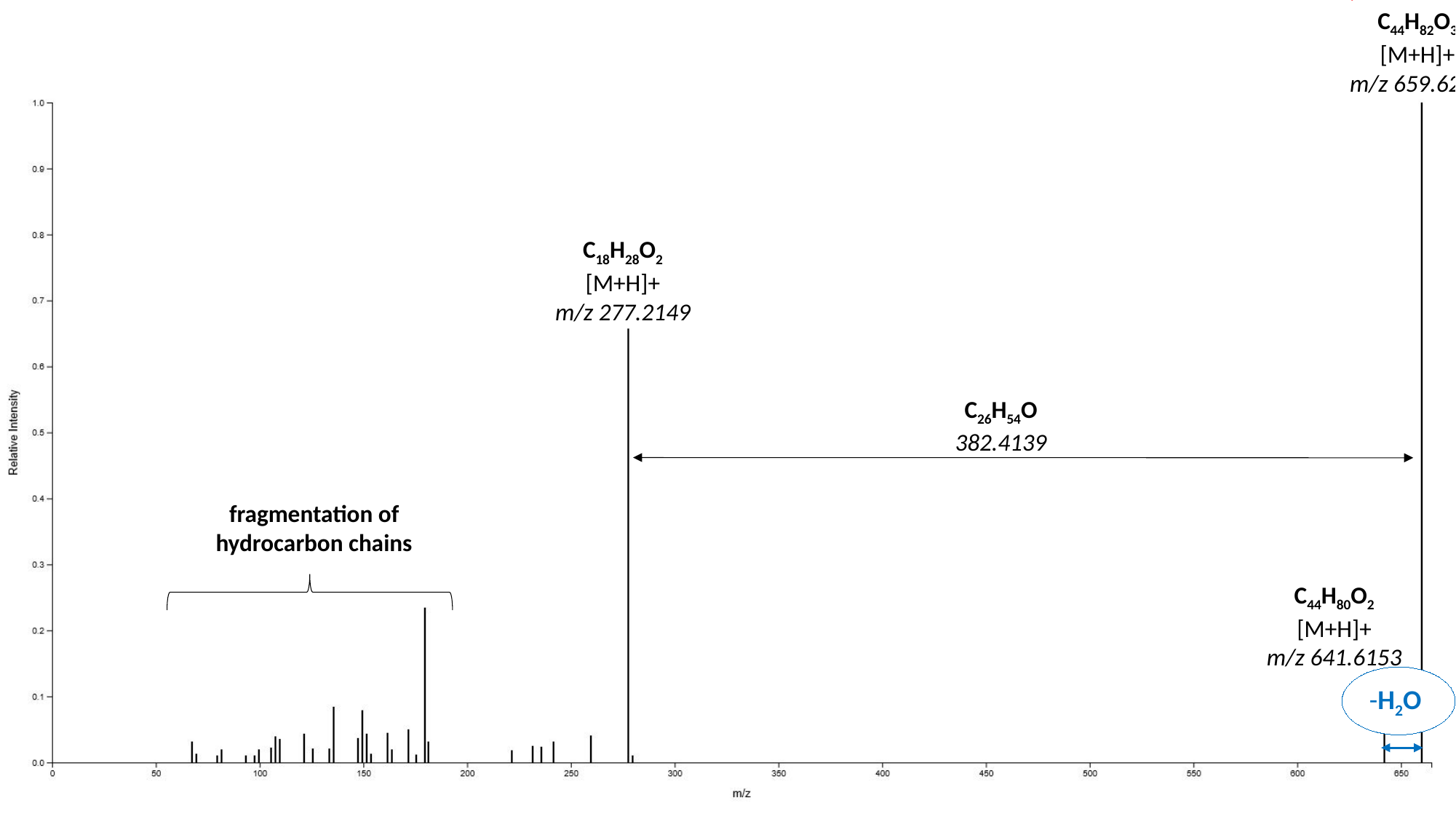

C44H82O3
[M+H]+
m/z 659.6288
hydroxy wax ester (26:0/18:3-O)
C18H28O2
[M+H]+
m/z 277.2149
C26H54O
382.4139
fragmentation of hydrocarbon chains
C44H80O2
[M+H]+
m/z 641.6153
-H2O

## Slide 27
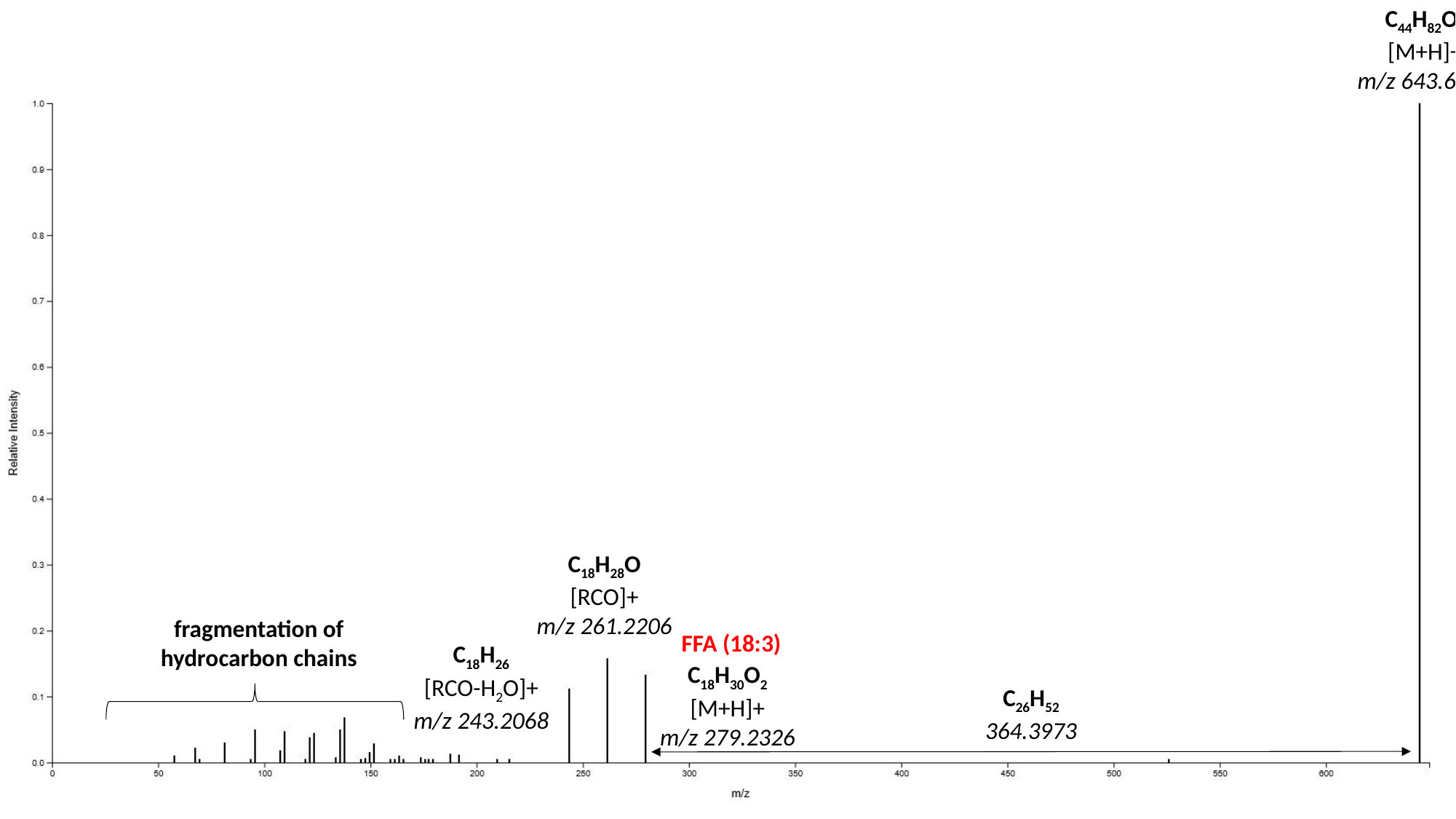

wax ester (26:0/18:3)
C44H82O2
[M+H]+
m/z 643.6299
C18H28O
[RCO]+
m/z 261.2206
fragmentation of hydrocarbon chains
FFA (18:3)
C18H26
[RCO-H2O]+
m/z 243.2068
C18H30O2
[M+H]+
m/z 279.2326
C26H52
364.3973

## Slide 28
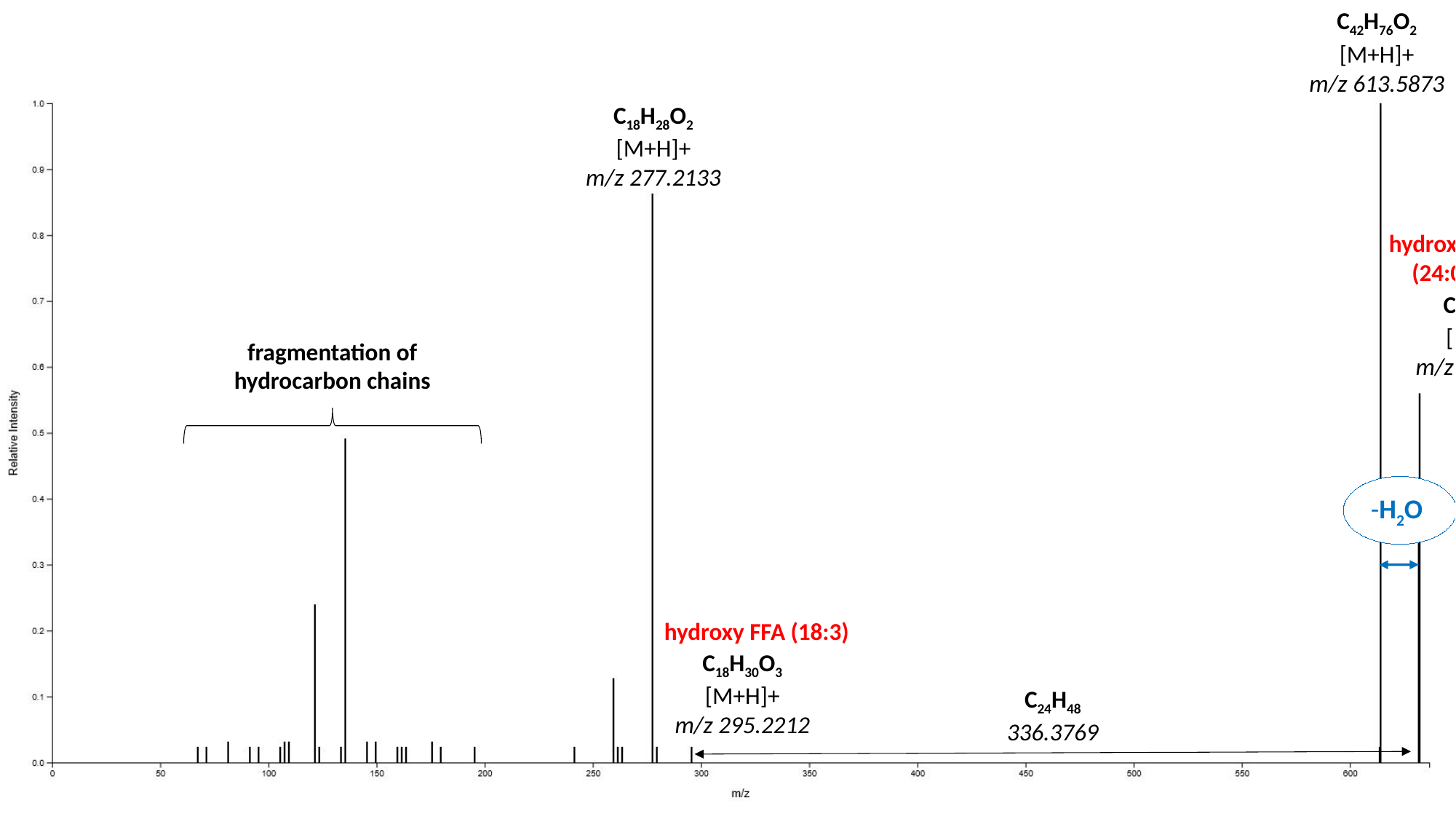

C42H76O2
[M+H]+
m/z 613.5873
C18H28O2
[M+H]+
m/z 277.2133
hydroxy wax ester (24:0/18:3-O)
C42H78O3
[M+H]+
m/z 631.5981
fragmentation of hydrocarbon chains
-H2O
hydroxy FFA (18:3)
C18H30O3
[M+H]+
m/z 295.2212
C24H48
336.3769
