# Supplementary material for: Metabolomics on Apple (Malus domestica) Cuticle—Search for Authenticity Markers
Source: Foods. 2024 Apr 24;13(9):1308. doi: 10.3390/foods13091308 (PMC11083494; doi:10.3390/foods13091308)
Supplement: Supplementary file 1 [file foods-13-01308-s001.zip › Supplementary_material_revize.pdf]

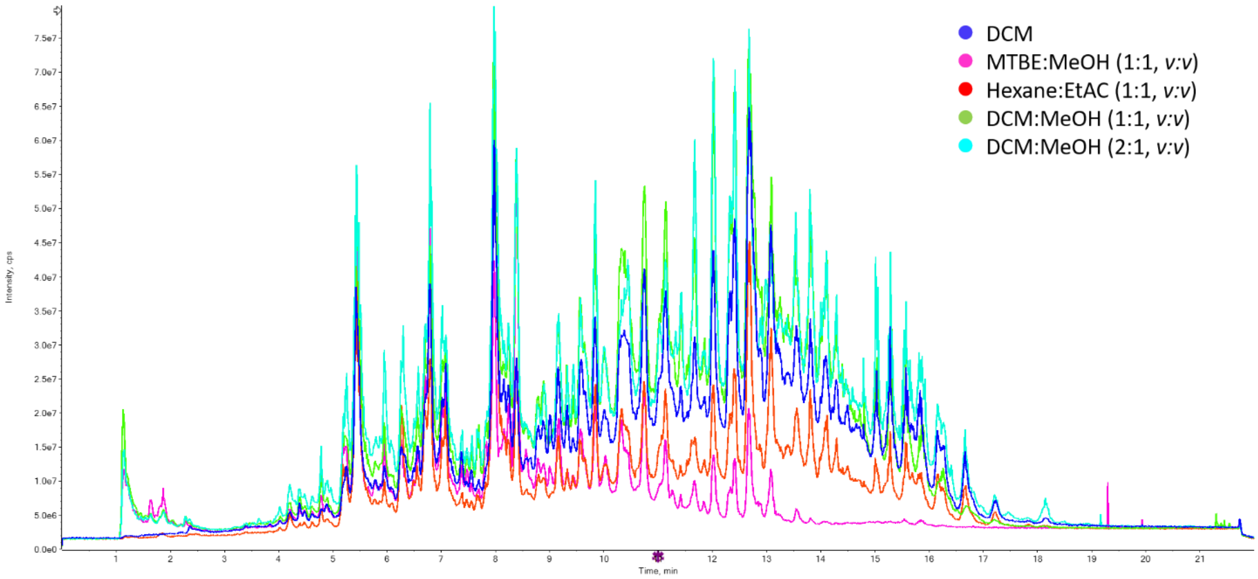

Figure S1: Total ion chromatograms (ESI+) showing intensities of compounds extracted by various solvent mixtures

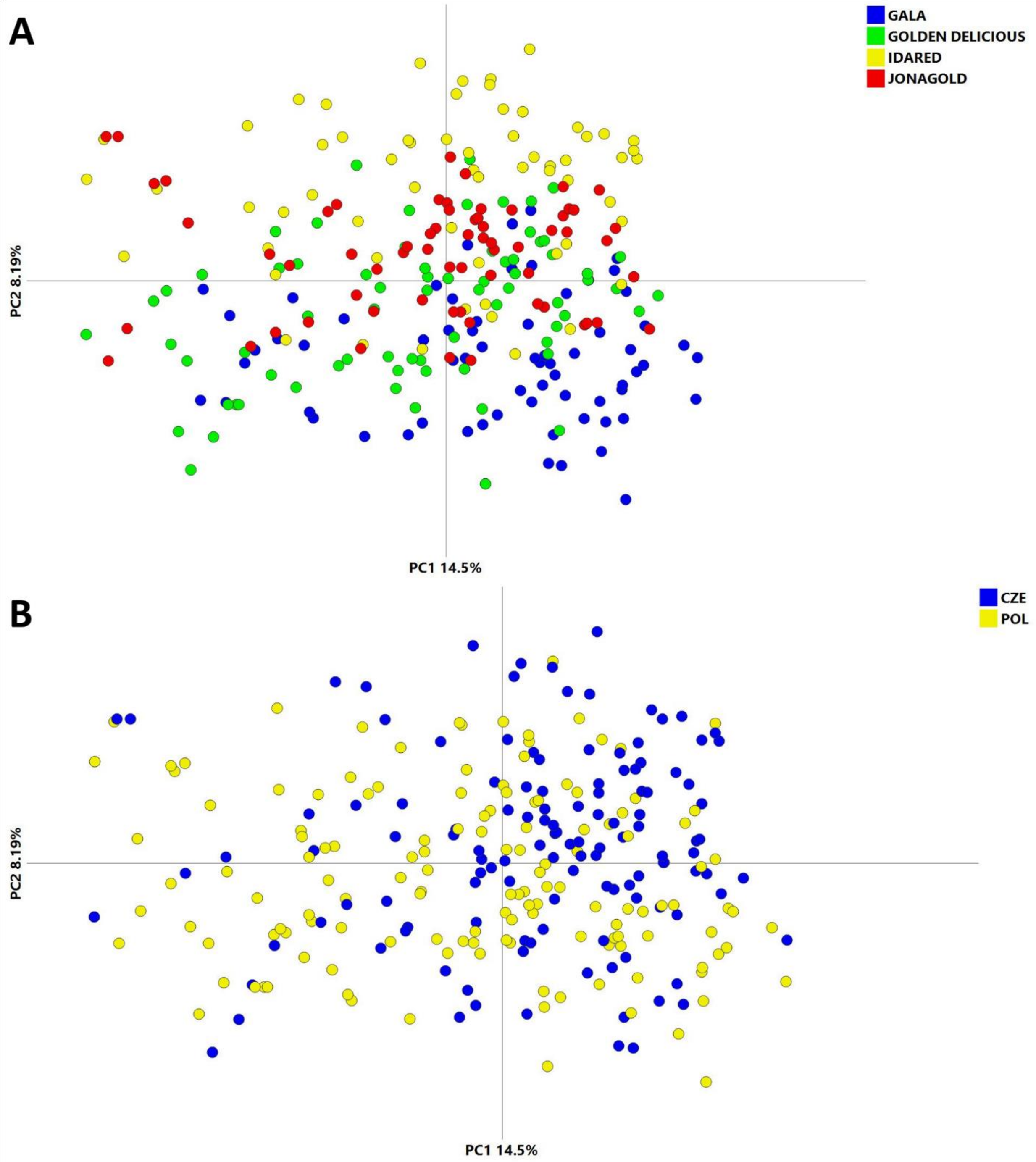

Figure S2: PCA Score plots of the complete dataset of ESI- features. Samples are coloured according to their cultivar (A) and geographical origin (*B*).

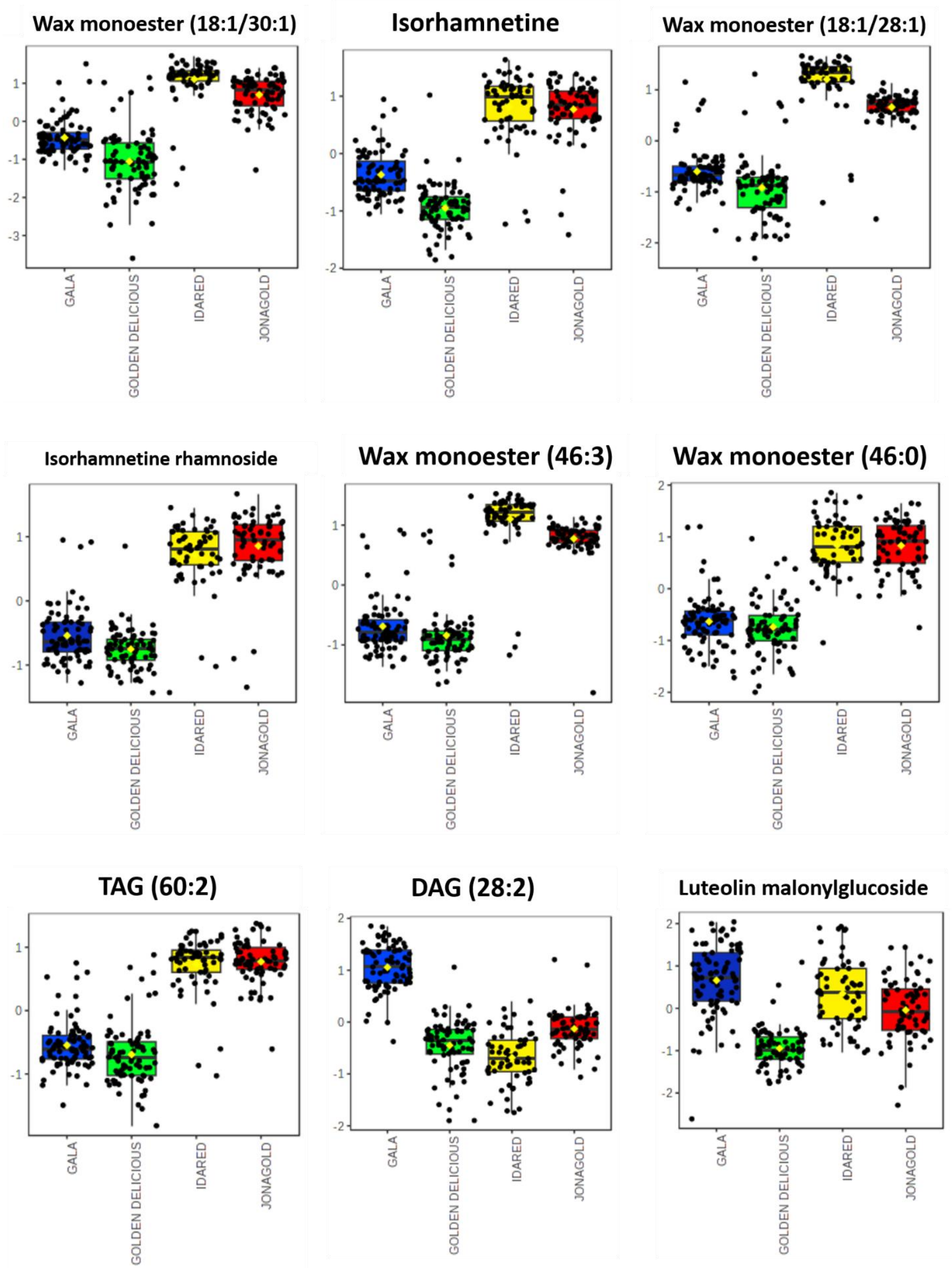
Wax monoester (18:1/30:1)
Isorhamnetine
Wax monoester (18:1/28:1)
Isorhamnetine rhamnoside
Wax monoester (46:3)
Wax monoester (46:0)
TAG (60:2)
DAG (28:2)
Luteolin malonylglucoside
GALA
GOLDEN DELICIOUS
IDARED
JONAGOLD

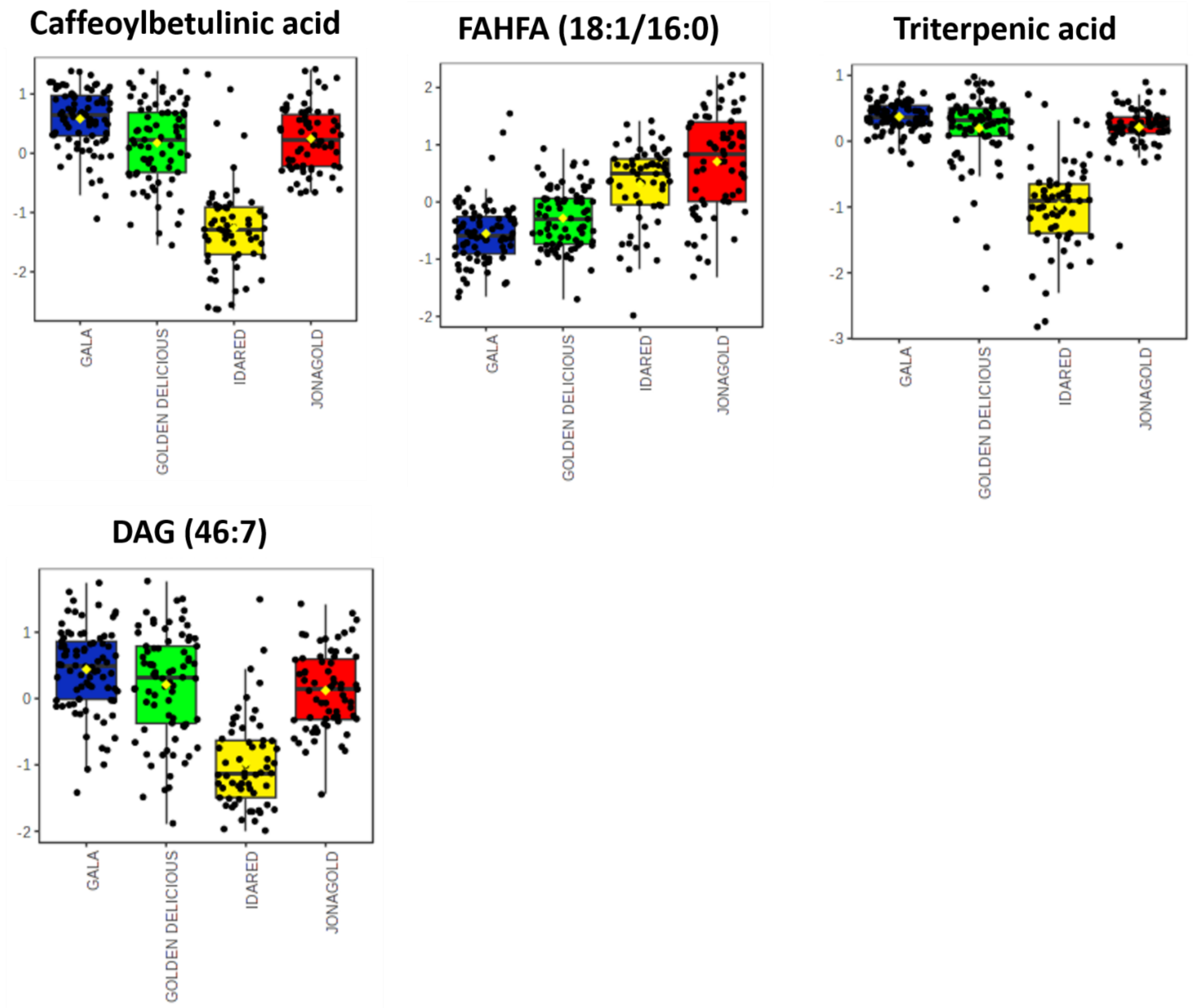

Figure S3: Boxplots of identified markers for classification of apple cultivar.

Table S1: Identification of metabolites used for geographical origin classification of apple cultivar 'Gala'. Markers are in descending order according to the AUC ROC value. The log2 FC value indicates whether the marker is increased in Czech samples (log2 FC > 0) or in Polish samples (log2 FC < 0).

Marker ion (m/z)	Retention time [min]	Adduct type	Elemental formula	Mass error [ppm]	Tentative identification	Univariate statistics parameters			Confidence level
						t-test FDR p-value	Log2 FC	AUC ROC	
635.6317	13.92	[M+H]+	$C_{42}H_{82}O_3$	-3.9	Hydroxy wax ester (24:0/18:1-O)	1.90E-08	0.78	0.91	2
661.6446	14.14	[M+H]+	$C_{44}H_{84}O_3$	-8	Hydroxy wax ester (26:0/18:2-O)	1.08E-05	0.61	0.9	2
135.0301	1.22	[M-H]-	$C_4H_8O_5$	5.6	Trihydroxybutanoic acid	1.05E-08	-2.83	0.87	3
619.634	13.48	[M+H]+	$C_{42}H_{82}O_2$	-8.6	Wax esters (42:1)	3.03E-08	0.86	0.86	3
607.5995	13.51	[M+H]+	$C_{40}H_{78}O_3$	-5.6	Hydroxy wax ester (40:1)	6.40E-07	0.73	0.85	3
637.6466	13.78	[M+H]+	$C_{42}H_{84}O_3$	-5.1	Hydroxy wax ester (26:0/16:0-O)	4.70E-07	1.09	0.84	2
663.6599	14.33	[M+H]+	$C_{44}H_{86}O_3$	-8.5	Hydroxy wax ester (26:0/18:1-O)	8.07E-05	0.6	0.84	2
609.6143	13.39	[M+H]+	$C_{40}H_{80}O_3$	-7	Hydroxy wax ester (24:0/16:0-O)	1.13E-06	1.02	0.83	2
521.524	14.33	[M+H]+	$C_{35}H_{68}O_2$	-11	Wax esters (26:0/9:1)	3.53E-05	0.53	0.82	2
659.6268	13.44	[M+H]+	$C_{44}H_{82}O_3$	-11.2	Hydroxy wax ester (26:0/18:3-O)	1.12E-06	0.68	0.82	2
619.6351	13.77	[M+H]+	$C_{42}H_{82}O_2$	-6.8	Wax esters (26:0/16:1)	2.26E-06	1.04	0.81	2
691.6917	14.73	[M+H]+	$C_{46}H_{90}O_3$	-7.5	Hydroxy wax ester (28:0/18:1-O)	3.51E-04	0.5	0.81	2
631.5949	13.02	[M+H]+	$C_{42}H_{78}O_3$	-12.7	Hydroxy wax ester (24:0/18:3-O)	4.68E-05	0.73	0.81	2
455.3456	5.17	[M+H]+	$C_{30}H_{46}O_3$	-15.2	Oxo ursenoic acid	6.76E-06	0.42	0.81	3
615.5967	13.74	[M+H]+	$C_{42}H_{78}O_2$	-18.4	Wax ester (24:0/18:3)	1.90E-05	0.74	0.8	2
661.6455	12.91	[M+H]+	$C_{44}H_{84}O_3$	-6.7	Hydroxy wax ester (24:0/18:2-O)	8.11E-06	0.84	0.8	2
265.2493	9.67	[M+H]+	$C_{18}H_{32}O$	-14.5	Linolenyl alcohol	8.69E-06	-1.12	0.79	3
287.2342	6.71	[M+H]+	$C_{20}H_{30}O$	-11.5	Vitamin A	2.43E-04	-0.89	0.79	3
471.3458	5.61	[M+H]+	$C_{30}H_{46}O_4$	-3.5	Oxohydroxy ursenoic acid	1.18E-04	0.44	0.79	3
673.6816	14.74	[M+H]+	$C_{46}H_{88}O_2$	-6.9	Wax ester (28:0/18:2)	1.19E-04	0.43	0.79	2
457.3627	6.54	[M+H]+	$C_{30}H_{48}O_3$	-11.9	Monohydroxy ursenoic acid	4.60E-04	0.27	0.79	3
549.5176	11.68	[M+H]+	$C_{36}H_{68}O_3$	-12.9	Hydroxy wax ester (36:2)	7.75E-05	0.41	0.78	3
263.2368	9.13	[M+H]+	$C_{18}H_{30}O$	-2.6	Farnesylacetol	9.36E-05	-1.02	0.77	3
318.2987	3.84	[M+H]+	$C_{18}H_{39}NO_3$	-6.7	Phytosphingosine	3.41E-05	0.75	0.77	3
453.3358	5.61	[M+H]+	$C_{30}H_{44}O_3$	-2.4	Oxodehydroursolic acid	2.13E-04	0.42	0.77	3
469.3625	6.73	[M+H]+	$C_{31}H_{48}O_3$	-12.1	Methyl oxooleanenoic acid	6.82E-04	0.34	0.77	3
615.6071	13.19	[M+H]+	$C_{42}H_{78}O_2$	-1.5	Wax ester (24:0/18:3)	9.98E-05	0.87	0.77	2
671.6644	13.95	[M+H]+	$C_{46}H_{86}O_2$	-9.2	Wax ester (28:0/18:3)	5.31E-05	0.71	0.77	2
673.6794	14.02	[M+H]+	$C_{46}H_{88}O_2$	-10.1	Wax ester (28:0/18:2)	4.17E-04	0.56	0.77	2

703.6281	15.83	[M+H]+	$C_{45}H_{82}O_5$	5.7	DAG (42:3)	6.02E-05	1.13	0.77	3
379.0965	1.34	[M+Na]+	$C_{16}H_{20}O_9$	-10.6	Feruloyl glucose	1.02E-04	1.11	0.76	3
475.1182	1.59	[M+Na]+	$C_{21}H_{24}O_{11}$	-7.2	Sieboldin	8.92E-04	0.6	0.76	3
525.3529	6.44	[M+Na]+	$C_{31}H_{50}O_5$	-5.1	Methyl ester euscaphic acid	9.20E-05	0.85	0.76	3
641.6169	15.19	[M+Na]+	$C_{42}H_{82}O_2$	-6.7	Wax ester (42:1)	4.38E-05	0.35	0.76	3
669.6464	13.82	[M+H]+	$C_{46}H_{84}O_2$	-12.7	Wax ester (28:0/18:4)	3.92E-04	0.62	0.76	2
683.6628	15.97	[M+Na]+	$C_{45}H_{88}O_2$	-7.9	Wax ester (45:1)	2.78E-04	0.76	0.76	3
471.3437	6.56	[M+H]+	$C_{30}H_{46}O_4$	-7.9	Oxohydroxy ursenoic acid	1.75E-04	0.25	0.75	3
453.3329	3.93	[M+H]+	$C_{30}H_{44}O_3$	-8.8	Oxodehydroursolic acid	3.66E-04	0.63	0.75	3
469.3327	5.91	[M+H]+	$C_{30}H_{44}O_4$	1.9	Dioxohydroxy ursenoic acid	4.58E-04	0.75	0.75	3
633.6124	13.74	[M+H]+	$C_{42}H_{80}O_3$	-9.7	Hydroxy wax ester (24:0/18:2-O)	2.92E-05	0.79	0.75	3

AUC – Area under curve, DAG - diacylglycerol FC – Fold Change, FDR – False discovery rate, ROC – Receiver operating characteristics

Table S2: Identification of metabolites used for geographical origin classification of apple cultivar 'Golden Delicious'. Markers are in descending order according to the AUC ROC value. The log2 FC value indicates whether the marker is increased in Czech samples (log2 FC > 0) or in Polish samples (log2 FC < 0).

Marker ion (m/z)	**Retention time [min]**	**Adduct type**	Elemental formula	**Mass error [ppm]**	**Tentative identification**	**Univariate statistics parameters**			**Confidence level**
						**t-test FDR p-value**	**Log2 FC**	**AUC ROC**	
671.6644	13.95	[M+H]+	$C_{46}H_{86}O_2$	-9.2	Wax ester (28:0/18:3)	1.30E-11	0.78	0.91	2
661.6455	12.91	[M+H]+	$C_{44}H_{84}O_3$	-6.7	Hydroxy wax ester (24:0/18:2-O)	5.98E-10	1.08	0.9	2
661.6455	13.58	[M+H]+	$C_{44}H_{84}O_3$	-6.6	Hydroxy wax ester (24:0/18:2-O)	2.78E-10	1.1	0.9	2
659.6295	12.78	[M+H]+	$C_{44}H_{82}O_3$	-7.1	Hydroxy wax ester (26:0/18:3-O)	4.99E-08	0.94	0.88	2
691.6893	14.19	[M+H]+	$C_{46}H_{90}O_3$	-10.8	Hydroxy wax ester (28:0/18:1-O)	2.92E-10	0.81	0.88	2
631.6307	14.14	[M+H]+	$C_{43}H_{82}O_2$	-13.7	Wax ester (43:2)	1.50E-08	0.68	0.87	3
643.6345	13.58	[M+H]+	$C_{44}H_{82}O_2$	-7.5	Wax ester (26:0/18:3)	4.11E-09	1.01	0.87	3
459.3801	5.89	[M+H]+	$C_{30}H_{50}O_3$	-8	Hydroxybetulin	1.91E-07	0.95	0.86	3
669.5425	12.12	[M+Na]+	$C_{41}H_{74}O_5$	-1.4	DAG (38:3)	2.96E-08	1.3	0.86	3
615.6071	13.19	[M+H]+	$C_{42}H_{78}O_2$	-1.4	Wax ester (24:0/18:3)	1.72E-07	1.24	0.85	2
647.557	12.13	[M+Na]+	$C_{39}H_{76}O_5$	-3.1	DAG (36:0)	1.68E-06	1.26	0.85	3
163.0745	10.88	[M+H]+	$C_{10}H_{10}O_2$	-8.9	Methyl cinnamate	7.18E-08	-1.63	0.84	3
183.0862	1.15	[M+H]+	$C_6H_{14}O_6$	-3.6	Sorbitol	9.54E-08	1.19	0.84	3
605.5795	11.99	[M+H]+	$C_{40}H_{76}O_3$	-12.8	Hydroxy wax ester (22:0/18:2-O)	5.83E-06	0.94	0.84	2
131.0465	1.16	[M-H]-	$C_4H_8N_2O_3$	6.3	Asparagine	8.20E-08	3.85	0.84	3
133.0628	12.05	[M+H]+	C_9H_8O	-19.3	Cinnamaldehyde	1.06E-06	0.67	0.83	3
425.3765	12.47	[M+H]+	$C_{30}H_{48}O$	-4.4	Lupeone	1.90E-07	-1.13	0.83	3
451.4453	14.44	[M+H]+	$C_{30}H_{58}O_2$	-13.9	Triacontenoic acid	2.38E-06	-1.73	0.83	3
487.4499	12.11	[M+H]+	$C_{33}H_{58}O_2$	-3.3	Wax ester (33:4)	1.12E-06	-0.96	0.83	3
165.0757	1.15	[M+H]+	$C_6H_{12}O_5$	-3.9	Fucose	6.22E-08	1.27	0.83	3
659.6288	13.76	[M+H]+	$C_{44}H_{82}O_3$	-8.2	Hydroxy wax ester (26:0/18:3-O)	2.26E-07	0.55	0.83	2
663.6599	14.33	[M+H]+	$C_{44}H_{86}O_3$	-8.5	Hydroxy wax ester (26:0/18:1-O)	6.63E-07	0.57	0.83	2
665.6752	14.16	[M+H]+	$C_{44}H_{88}O_3$	-9	Hydroxy wax ester (44:0)	4.72E-07	0.64	0.83	3
691.6917	14.73	[M+H]+	$C_{46}H_{90}O_3$	-7.5	Hydroxy wax ester (28:0/18:1-O)	2.32E-06	0.27	0.83	2
711.585	11.77	[M+Na]+	$C_{44}H_{80}O_5$	-7.6	DAG (41:3)	5.56E-07	-1.38	0.83	3
445.3968	13.46	[M+H]+	$C_{30}H_{52}O_2$	-17.4	Wax ester (30:4)	1.43E-06	-1.57	0.82	3
659.6268	13.44	[M+H]+	$C_{44}H_{82}O_3$	-11.2	Hydroxy wax ester (26:0/18:3-O)	1.87E-06	0.44	0.82	2
299.0562	2.86	[M-H]-	$C_{16}H_{12}O_6$	2.1	Luteolin methyl ether	5.75E-07	-1.82	0.82	3
265.2524	11.32	[M+H]+	$C_{18}H_{32}O$	-2.6	Linolenyl alcohol	3.45E-06	-1.17	0.81	3
457.3661	7.28	[M+H]+	$C_{30}H_{48}O_3$	-4.6	Monohydroxy ursenoic acid	1.51E-06	0.1	0.81	3

635.6317	13.92	[M+H]+	$C_{42}H_{82}O_3$	-3.9	Hydroxy wax ester (24:0/18:1-O)	5.44E-06	0.28	0.81	2
673.6816	14.74	[M+H]+	$C_{46}H_{88}O_2$	-6.9	Wax ester (28:0/18:2)	7.60E-06	0.2	0.81	2
675.5843	12.6	[M+Na]+	$C_{41}H_{80}O_5$	-9	DAG (38:0)	8.74E-07	1.14	0.81	3
853.7221	15.53	[M+Na]+	$C_{53}H_{98}O_6$	-4.8	TAG (40:2)	3.75E-07	-1.41	0.81	3
875.7018	14.04	[M+Na]+	$C_{55}H_{96}O_6$	-9.9	TAG (52:5)	2.86E-05	0.66	0.81	3
877.7224	15.24	[M+Na]+	$C_{55}H_{98}O_6$	-4.2	TAG (52:4)	1.48E-06	-1.65	0.81	3
181.0744	1.15	[M-H]-	$C_6H_{14}O_6$	17.6	Galacticol	5.16E-07	0.83	0.81	3
263.2368	9.13	[M+H]+	$C_{18}H_{30}O$	-2.8	Farnesylacetol	1.04E-04	-1.31	0.8	3
133.0642	10.42	[M+H]+	C_9H_8O	-8.9	Cinnamaldehyde	2.39E-05	-1.27	0.8	3
193.1605	9.13	[M+H]+	$C_{13}H_{20}O$	6.6	Damascone	2.07E-06	-1.37	0.8	3
443.3858	13.44	[M+H]+	$C_{30}H_{50}O_2$	-7.1	Uvaol	7.36E-06	-1.27	0.79	3
471.3458	5.61	[M+H]+	$C_{30}H_{46}O_4$	-3.5	Hydroxyoxooleane noic acid	4.97E-05	0.37	0.79	3
643.6299	14.14	[M+H]+	$C_{44}H_{82}O_2$	-14.6	Wax ester (26:0/18:3)	1.34E-05	0.18	0.79	2
427.3927	7.83	[M+H]+	$C_{30}H_{50}O$	-3.1	Amyrin	2.32E-05	0.44	0.78	3
445.3971	10.15	[M+H]+	$C_{30}H_{52}O_2$	-16.8	Wax ester (30:4)	9.40E-03	-2.15	0.78	3
445.3997	13.72	[M+H]+	$C_{30}H_{52}O_2$	-10.9	Wax ester (30:4)	2.48E-05	-1.24	0.78	3
453.3358	5.61	[M+H]+	$C_{30}H_{44}O_3$	-2.4	Oxodehydroursolic acid	8.40E-05	0.37	0.78	3
593.5082	10.9	[M+H]+	$C_{37}H_{68}O_5$	-10.6	DAG (34:2)	2.11E-05	-1.63	0.78	3
631.5949	13.02	[M+H]+	$C_{42}H_{78}O_3$	-12.7	Hydroxy wax ester (24:0/18:3-O)	3.94E-05	0.65	0.78	2
661.6446	14.14	[M+H]+	$C_{44}H_{84}O_3$	-8	Hydroxy wax ester (26:0/18:2-O)	3.26E-05	0.19	0.78	2
635.4605	10.35	[M+Na]+	$C_{39}H_{64}O_5$	-7.4	DAG (36:6)	3.96E-05	1.86	0.78	3
685.5684	13.44	[M+Na]+	$C_{42}H_{78}O_5$	-9.2	DAG (38:2)	9.87E-05	-1.23	0.78	3
275.0891	1.8	[M+H]+	$C_{15}H_{14}O_5$	-10.4	Phloretin	3.23E-05	0.43	0.77	3
443.3849	7.64	[M+H]+	$C_{30}H_{50}O_2$	-9.1	Wax ester (30:5)	3.72E-05	0.32	0.77	3
543.4717	12.4	[M+H]+	$C_{36}H_{62}O_3$	-11	Hydroxy wax ester (36:5)	2.23E-05	-1.17	0.77	3
609.6143	13.39	[M+H]+	$C_{40}H_{80}O_3$	-7	Hydroxy wax ester (24:0/16:0-O)	6.61E-05	0.55	0.77	3
619.6351	13.77	[M+H]+	$C_{42}H_{82}O_2$	-6.7	Wax ester (26:0/16:1)	1.40E-05	0.59	0.77	2
615.4914	11.35	[M+Na]+	$C_{37}H_{68}O_5$	-8.2	DAG (34:2)	5.68E-05	0.63	0.77	3
699.5872	12.84	[M+Na]+	$C_{43}H_{80}O_5$	-4.6	DAG (40:2)	2.73E-04	0.7	0.77	3
133.0139	1.25	[M-H]-	$C_4H_6O_5$	1.5	Malic acid	3.65E-05	2.11	0.77	3
209.0666	1.2	[M-H]-	$C_7H_{14}O_7$	2.2	Heptulose	5.58E-05	1.32	0.77	3
447.4142	10.22	[M+H]+	$C_{30}H_{54}O_2$	-13.5	Wax ester (30:3)	7.10E-05	-1.29	0.76	3
455.3494	5.84	[M+H]+	$C_{30}H_{46}O_3$	-6.9	Oxooleanolic acid	2.98E-05	0.04	0.76	3
663.5863	12.23	[M+H]+	$C_{42}H_{78}O_5$	-9.7	DAG (38:2)	1.47E-04	-1.35	0.76	3
495.3431	5.41	[M+Na]+	$C_{30}H_{48}O_4$	-3.9	Hydroxyursolic acid	4.54E-05	0.24	0.76	3
650.5706	11.22	[M+NH4]+	$C_{40}H_{72}O_5$	-2.7	DAG (36:3)	2.52E-05	-1.36	0.76	3
198.0964	1.15	[M+NH4]+	$C_6H_{12}O_6$	-6.7	Mannose	2.32E-04	1.1	0.76	3
631.5981	13.29	[M+H]+	$C_{42}H_{78}O_3$	-7.7	Hydroxy wax ester (24:0/18:3-O)	2.59E-03	0.21	0.75	2
613.4818	8.72	[M+Na]+	$C_{37}H_{66}O_5$	1.7	DAG (34:3)	2.75E-04	0.81	0.75	3
685.5693	10.25	[M+Na]+	$C_{42}H_{78}O_5$	-7.9	DAG (38:2)	1.04E-04	-1.12	0.75	3
851.7066	13.8	[M+Na]+	$C_{53}H_{96}O_6$	-4.5	TAG (40:3)	3.96E-05	-0.93	0.75	3

AUC – Area under curve, DAG - diacylglycerol FC – Fold change, FDR – False discovery rate, ROC – Receiver operating characteristics, TAG – triacylglycerol

Table S3: Identification of metabolites used for geographical origin classification of apple cultivar 'Idared'. Markers are in descending order according to the AUC ROC value. The log2 FC value indicates whether the marker is increased in Czech samples (log2 FC > 0) or in Polish samples (log2 FC < 0).

Marker ion (m/z)	**Retention time [min]**	**Adduct type**	Elemental formula	**Mass error [ppm]**	**Tentative identification**	**Univariate statistics parameters**			**Confidence level**
						**t-test FDR p-value**	**Log2 FC**	**AUC ROC**	
167.0709	1.74	[M+H]+	C9H10O3	0.5	Ethyl salicylate	1.37E-05	-2.82	0.82	3
225.0587	1.15	[M+HCOO]-	C6H12O6	10.4	Mannose	1.04E-05	1.25	0.82	3
561.1335	1.45	[M+H]+	C30H24O11	-11	Biflavonoid	1.31E-04	1.69	0.78	3
209.0666	1.2	[M-H]-	C7H14O7	2.2	Heptulose	4.21E-05	1.18	0.78	3
353.0853	1.48	[M-H]-	C16H18O9	5.6	Chlorogenic acid	3.18E-04	1.06	0.76	3
131.0465	1.16	[M-H]-	C4H8N2O3	6.2	Asparagine	4.54E-04	1.15	0.75	3

AUC – Area under curve, FC – Fold change, FDR – False discovery rate, ROC – Receiver operating characteristics

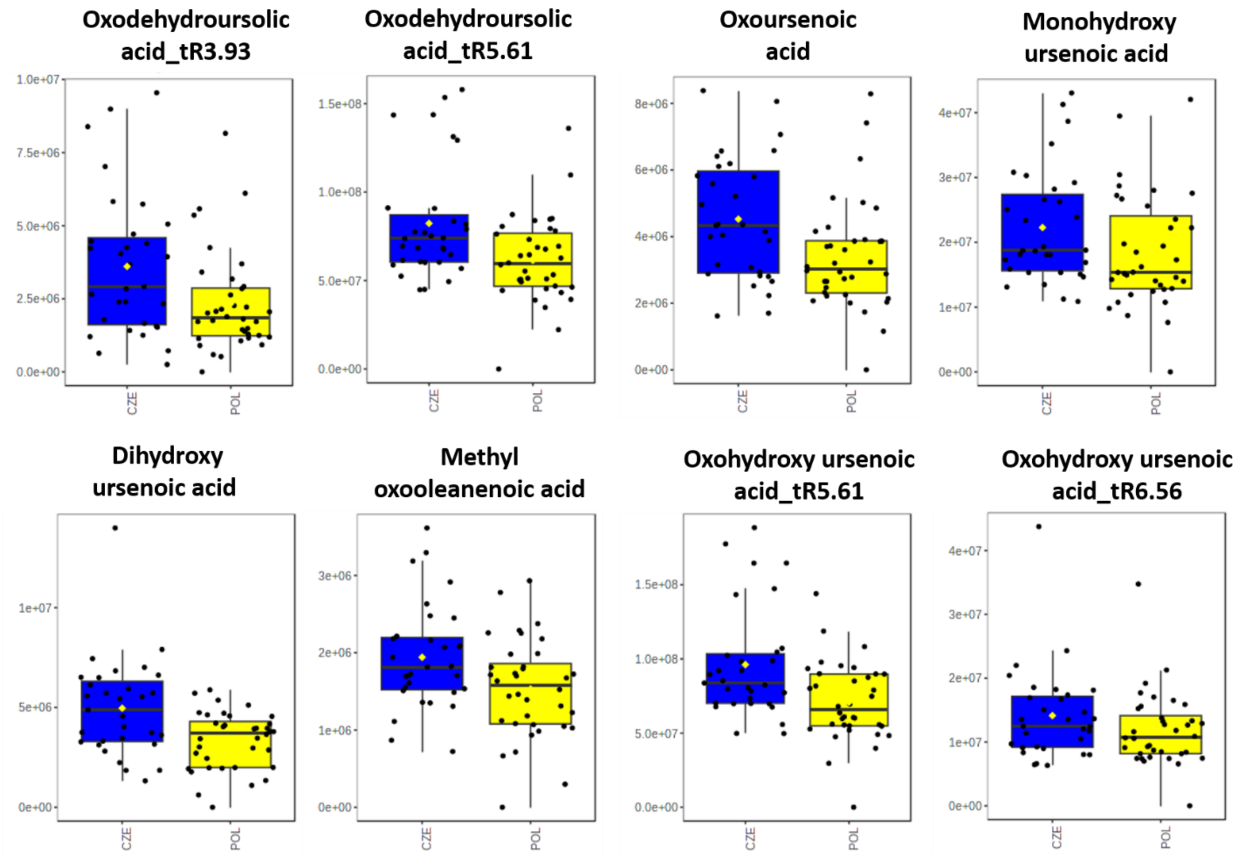

Figure S4: Boxplots of markers for classification of geographical origin for cultivar 'Gala' identified as triterpenic ursenoic acids and their derivatives and precursors.

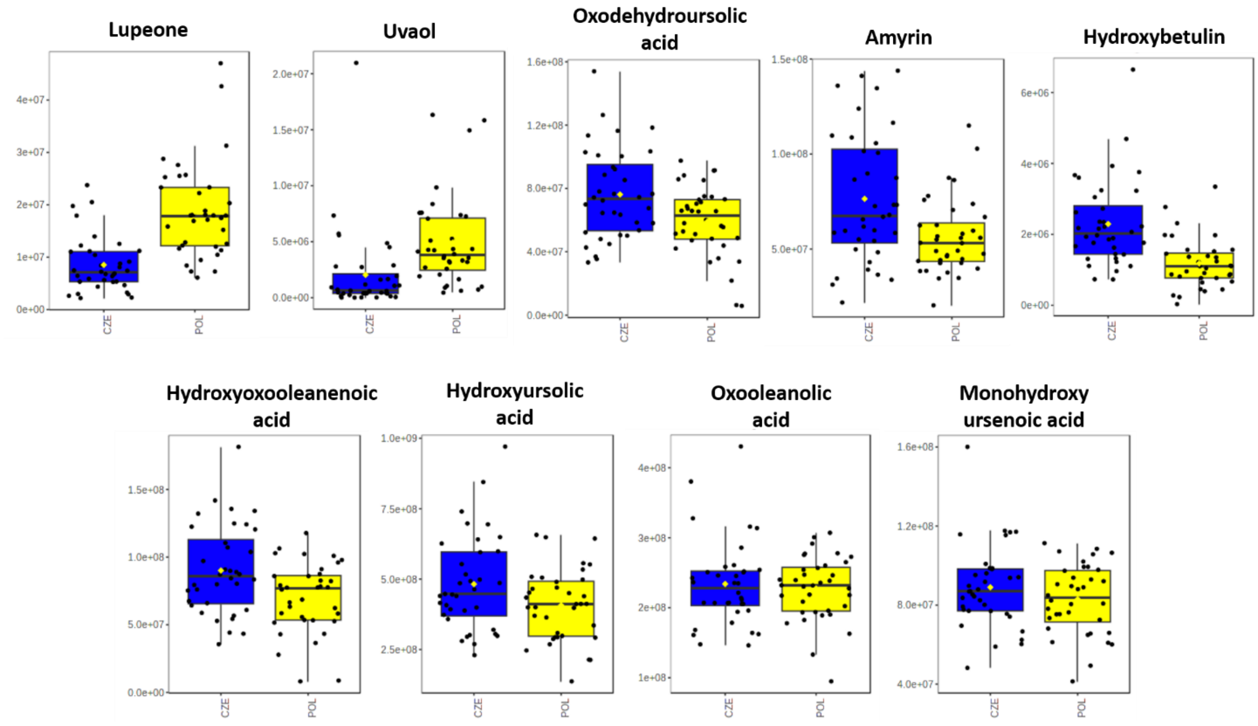

Figure S5: Boxplots of markers for classification of geographical origin for cultivar 'Golden Delicious' identified as triterpenic ursenoic acids and their derivatives and precursors.
